# Identification of Mycobacterium tuberculosis Peptides in Serum Extracellular Vesicles from Persons with Latent Tuberculosis Infection

**DOI:** 10.1128/JCM.00393-20

**Published:** 2020-05-26

**Authors:** Carolina Mehaffy, Nicole A. Kruh-Garcia, Barbara Graham, Leah G. Jarlsberg, Charis E. Willyerd, Andrey Borisov, Timothy R. Sterling, Payam Nahid, Karen M. Dobos

**Affiliations:** a Department of Microbiology, Immunology and Pathology, Colorado State University, Fort Collins, Colorado, USA; b Proteomic and Metabolomics Facility, Colorado State University, Fort Collins, Colorado, USA; c Division of Pulmonary and Critical Care Medicine, University of California San Francisco, Zuckerberg San Francisco General, San Francisco, California, USA; d Clinical Data Science Associates, LLC, Fort Collins, Colorado, USA; e Division of Tuberculosis Elimination, Centers for Disease Control and Prevention, Atlanta, Georgia, USA; f Department of Medicine, Vanderbilt University Medical Center, Nashville, Tennessee, USA; Carter BloodCare & Baylor University Medical Center

**Keywords:** biomarker, LTBI, MRM-MS, tuberculosis

## Abstract

Identification of biomarkers for latent Mycobacterium tuberculosis infection and risk of progression to tuberculosis (TB) disease are needed to better identify individuals to target for preventive therapy, predict disease risk, and potentially predict preventive therapy efficacy. Our group developed multiple reaction monitoring mass spectrometry (MRM-MS) assays that detected M. tuberculosis peptides in serum extracellular vesicles from TB patients. We subsequently optimized this MRM-MS assay to selectively identify 40 M. tuberculosis peptides from 19 proteins that most commonly copurify with serum vesicles of patients with TB. Here, we used this technology to evaluate if M. tuberculosis peptides can also be detected in individuals with latent TB infection (LTBI). Serum extracellular vesicles from 74 individuals presumed to have latent M. tuberculosis infection (LTBI) based on close contact with a household member with TB or a recent tuberculin skin test (TST) conversion were included in this study. Twenty-nine samples from individuals with no evidence of TB infection by TST and no known exposure to TB were used as controls to establish a threshold to account for nonspecific/background signal. We identified at least one of the 40 M. tuberculosis peptides in 70 (95%) individuals with LTBI. A single peptide from the glutamine synthetase (GlnA1) enzyme was identified in 61/74 (82%) individuals with LTBI, suggesting peptides from M. tuberculosis proteins involved in nitrogen metabolism might be candidates for pathogen-specific biomarkers for detection of LTBI. The detection of M. tuberculosis peptides in serum extracellular vesicles from persons with LTBI represents a potential advance in the diagnosis of LTBI.

## Abstract (precis)

Identification of biomarkers for latent Mycobacterium tuberculosis infection and risk of progression to tuberculosis (TB) disease are needed to better identify individuals to target for preventive therapy, predict disease risk, and potentially predict preventive therapy efficacy. Our group developed multiple reaction monitoring mass spectrometry (MRM-MS) assays that detected M. tuberculosis peptides in serum extracellular vesicles from TB patients. We subsequently optimized this MRM-MS assay to selectively identify 40 M. tuberculosis peptides from 19 proteins that most commonly copurify with serum vesicles of patients with TB.

## INTRODUCTION

Tuberculosis (TB) is the leading cause of death due to single infectious agent (1). Public health control of the disease is difficult for several reasons, including lack of an effective vaccine, diagnostic and treatment delays, and the emergence of multidrug-resistant Mycobacterium tuberculosis strains. About one quarter of the global population (1.7 billion people) is estimated to have latent TB infection (LTBI) (1, 2). Approximately 5 to 10% of these individuals will progress to TB disease (3). Individuals with LTBI harbor the bacteria in their lungs but do not present any clinical symptoms and are not contagious. However, they are at risk of developing TB, including the classic symptoms of the disease such as productive chronic cough, night sweats, and weight loss. In contrast to LTBI, individuals with TB usually have M. tuberculosis in their sputum, resulting in spread of the disease. Treatment of individuals with LTBI has been effective in preventing TB, thus reducing the risk of transmission (4–6). However, some conditions, such as malnutrition, HIV infection, and age of <5 years old increase the risk of progression to TB disease. In the United States, most TB cases are attributed to reactivation of LTBI (7, 8). Biomarkers of LTBI could be used to identify individuals in need of preventive treatment, and thereby decrease TB risk.

LTBI is currently diagnosed by the tuberculin skin test (TST) or interferon gamma release assays (IGRA) (9–14). Both tests rely on an adequate host immune response and do not differentiate between LTBI and TB (10, 11, 15). Thus, novel diagnostic methods are needed to clearly identify individuals with LTBI, especially in high-risk groups in which the TST and IGRA may have low sensitivity, such as those living with HIV, cancer, diabetes, or other immunosuppressed conditions. Identifying biomarkers of infection may lead to faster and more reliable diagnostic methods for LTBI. Several host biomarkers have been proposed to differentiate between LTBI and TB (16–21), as well as to indicate the risk of disease progression (22, 23). Mycobacterial biomarkers of infection have been less studied, but evidence from our group and others indicates they could also be exploited for diagnostics purposes (24–26).

Extracellular vesicles (EVs) include exosomes, microvesicles, and apoptotic bodies. EVs are released from all nucleated cells and can be purified from different biofluids such as blood plasma, blood serum, and urine. Previous studies have demonstrated that *Mycobacterium* cell components, including RNA, lipids, and proteins, are found in EVs released from infected cells (27–34, 76). In a proof-of principle study, our group demonstrated that M. tuberculosis proteins can be detected in serum EVs isolated from individuals with LTBI and active TB (24). While we used both shotgun proteomics as well as MRM-MS in that initial study, there were several factors that hindered our conclusions. First, individuals with LTBI were recruited when they presented to the hospital with symptoms of TB but did not have laboratory evidence of TB. Thus, it is possible that factors associated with other diseases may have been at play and could have contributed to the EVs content in these subjects. Second, the MRM-MS assays developed in our first study did not include synthetic labeled peptide standards, and thus the detection of each peptide was neither validated nor quantitative. Our first study, however, provided the platform needed to evaluate exosomes as potential carriers of M. tuberculosis biomarkers. In our subsequent study, we optimized the MRM-MS assays to include additional targets, as well as to allow for monitoring of synthetic labeled standards. We then proceeded to validate these assays in a well-defined cohort of TB patients from geographical regions of high TB incidence, along with healthy controls (25). We demonstrated that MRM-MS technology detected femtomolar concentrations of M. tuberculosis peptides in a complex sample, such as serum EVs isolated from TB patients (25).

In the present study, we used the same technology and methodology to identify the M. tuberculosis peptide combinations and frequency of detection in serum EVs isolated from individuals with LTBI before treatment, establishing the groundwork for future studies to determine if these biomarkers may be used as part of prognostic tools to predict LTBI treatment success and/or progression to TB disease.

## MATERIALS AND METHODS

### Ethics statement.

This project was reviewed by the Institutional Review Board (IRB) at Colorado State University (CSU) and deemed human subject research exempt (IRB ID number 015-10H). The manipulation of human serum samples was approved by the Institutional Biosafety Committee at CSU (IBC PARF number 11-060B). Samples from persons with LTBI were obtained from participants in TB Trials Consortium Study 26, which was approved by the CDC IRB (protocol number 3041) and by IRBs at each of the participating clinical sites. Healthy control samples were kindly donated by David Lewinsohn at Oregon Health and Science University (IRB protocol e0186).

### Samples and study design.

**LTBI samples.** Serum samples (*n* = 74) from individuals selected for LTBI treatment were obtained from the Centers for Disease Control and Prevention’s TB Trials Consortium PREVENT TB Study (TBTC Study 26) (5). All participants in this substudy were enrolled from one of five study sites within the United States as follows: site 14, Maryland (*n* = 5); site 20, North Texas, (*n* = 24); site 22, Colorado (*n* = 14); site 63, South Texas (*n* = 19); and site 70, Tennessee (*n* = 12). All participants were diagnosed with LTBI based on a positive tuberculin skin test, and were either a household contact of a TB patient or had a documented skin test conversion from negative to positive within the previous 2 years. Based on these factors, participants were considered high risk for progression to TB (5). All samples were collected prior to treatment initiation for LTBI. None of the individuals in this substudy progressed to TB in the 33-month follow-up period. All samples were stored at –80°C until processed for exosome purification.

**Healthy control samples.** Banked serum samples from individuals with negative TSTs and/or negative in-house IGRAs were kindly donated by David Lewinsohn (*n* = 29; Oregon Health and Sciences University) and were collected using the identical protocol as the TBTC Study 26 samples. All samples were stored at –80°C until processed for exosome purification, which was performed as previously described (25). See below for details.

### Sample processing.

Personal identifiers were removed from all serum samples, and samples were then assigned an unsorted code and randomly selected prior to processing. EVs were isolated as previously described (24, 25). One hundred microliters of serum samples was centrifuged at 3,000 × *g* for 15 min to remove whole cells and cellular debris. Twenty-five microliters of Exoquick (System Biosciences, Palo Alto, CA) was added to the cleared serum, mixed well, and incubated overnight at 4°C. EVs were pelleted by centrifugation at 1,500 × *g* for 30 min. The supernatant depleted of EVs was discarded and the pellet was resuspended in 100 μl phosphate-buffered saline (PBS) (Corning, Corning, NY). The protein content of the EVs was quantified by micro bicinchoninic acid assay (Thermo Scientific Pierce, Rockford, IL). Thirty micrograms of each purified EV sample was loaded into a NuPAGE 4 to 20% gradient Tris-glycine gel (Invitrogen, Carlsbad, CA) and resolved for 5 min at 200 V to remove residual Exoquick polymer from the protein. The region of the gel containing EVs was excised, cut into 1-mm pieces, and digested with trypsin as previously described (24, 30). The extracted peptides were dried and resuspended in a solution of 5% acetonitrile, 0.1% formic acid, and 94.9% water. Isotope-labeled standards for each peptide (10 nM) (New England Peptides, Gardner, MA) (listed in Table 1) and iRT (Biognosys, Schlieren, Switzerland) retention time standards were spiked into each digest sample. The final concentration of the digested EVs in each sample was roughly 1 μg/μl.

**TABLE 1:** List of peptides targeted for identification in serum extracellular vesicles from individuals with latent tuberculosis infection (LTBI)

Method 1		Method 2	
Protein	Peptide^*a*^	Protein	Peptide
Ag85c (Rv0129c)	**VQF**QGGGPHAVYLLDGLR	DnaK (Rv0350)	**TTP**SIVAFAR
	**NDP**MVQIPR		**ITQ**DLLDR
	**FLE**GLTLR	Ag85B (Rv1886c)	**PGL**PVEYLQVPSPSMGR
GclB (Rv1837c)	**VVA**DLTPQNQALLNAR.D		**AAD**MWGPSSDPAWER
	**FAL**NAANAR	Cfp10 (Rv3874)	**QEL**DEISTNIR
	**NYT**APGGGQFTLPGR		
Mpt32 (Rv1860)	**TTG**DPPFPGQPPPVANDT	EsxA (Rv3875)	**LAA**AWGGSGSEAYQGVQQK
	**LYA**SAEATDSK		
Mpt64 (Rv1980c)	**SLE**NYIAQTR	GlnA1 (Rv2220)	**SVF**DDGLAFDGSSIR
	**FLS**AATSSTPR		**GGY**FPVAPNDQYVDLR
HspX (Rv2031c)	**AEL**PGVDPDK	MrsA (Rv3441c)	**YVL**EELR
	**TVS**LPVGADEDDIK		**TAV**EQAAAELGDTGR
Cfp2 (Rv2376c)	**GSL**VEGGIGGTEAR	PpiA (Rv0009)	**IAL**FGNHAPK
	**SLA**DPNVSFANK		**VIQ**GFMIQGGDPTGTGR
SahH (Rv3248c)	**GVT**EETTTGVLR		**HTI**FGEVIDAESQR
	**IHV**EALGGHLTK	AcpM (Rv2244)	**IPD**EDLAGLR
GroES (Rv3418c)	**DVL**AVVSK		**TVG**DVVAYIQK
	**RIP**LDVAEGDTVIYSK		**LEE**ENPEAAQALR
BfrB (Rv3841)	**EAL**ALALDQER	Ag85A (Rv3804c)	**NDP**LLNVGK
	**AGA**NLFELENFVAR		**FLE**GFVR
		GarA (Rv1827)	**FLL**DQAITSAGR
			**LVF**LTGPK

a The first three letters of each peptide (bold and underlined) are used for reference throughout the manuscript.

MRM-MS methods 1 and 2 were carried out as previously described (25). See Table 1 for a list of targets.

### MRM-MS data analysis.

Data acquired using the Xevo TQ-S mass spectrometer (Waters Corporation, Milford, MA) were exported into Skyline Daily (64 bit) (35). Skyline files were visually inspected for retention time and peak-boundary consistency. If needed, peak boundaries of the heavy-labeled standard peptides were manually corrected such that each peptide was consistent among all samples. The peak areas for each peptide/transition ion, total area ratio, and library dot-product (DotP) value for each sample were exported into Excel. Base SAS v9.4 was utilized to automate analysis and processing of the Skyline output data (see Supplemental Material 1). The output reports the normalized total peak average (nTPA) for each peptide with a delta DotP value equal to or less than 0.1. Higher delta DotP values were associated with significant deviation in fragmentation pattern from the isotopic standard and therefore the native peaks were disqualified and reported with a zero value. As isotopic standards are requisite for confident identification of the native peak, any peptide without a confident standard peak was removed from the analysis. The threshold of positivity was calculated as the 95^th^ percentile of the nTPA for each peptide signal from healthy controls using GraphPad Prism (v.6 and v.8) (see Supplemental Material 2). This threshold was then applied to the nTPA signal from subjects with LTBI to determine if the peptide was detected or not. If detected, the nTPA numeric value was used for further statistical analysis.

### Statistical analysis.

Descriptive statistics for demographics of LTBI individuals and MRM data were analyzed in R (version 3.5.1 [2018-07-02]). Signal from the heavy-labeled spiked internal standard was not observed for some peptides in some samples. Since we relied on the detection of heavy peptides to confidently identify the native (light) counterparts, as well as to normalize the total peak average (TPA) of each peptide, cases in which the internal standard was not detected were labeled as unknowns. Number of peptides detected and number of unknowns were modeled separately with linear models to determine whether or not any demographic traits might have a significant association with the number of peptides detected.

Traits that might be similar within a group were evaluated using classification trees with the complete data set. Because classification trees often fit well to specific data sets, but are not readily applied to new data, these results were reported as descriptive statistics, and no claim regarding statistical significance was made. All traits were individually tested for independence from categories (i.e., presence of peptides and presence of unknown) with a Fisher’s exact test.

A correlation matrix was calculated for the 31 of the 40 peptides that had at least one detectable value. Scatterplots of peptide concentrations were drawn to systematically review: (i) concentration distributions for each peptide, and (ii) relationships between paired peptides. Missing values were imputed as the concentration median, and a nonparametric Spearman method was used to calculate correlation coefficients and *P* values for each paired peptide comparison. Peptide pairs in the correlation matrix were ordered based on distance statistics using the hierarchical clustering method in R corrplot package (36) and the ordered correlations were tested to identify clusters. Further cluster analysis was done by calculating a distance matrix of dissimilarity equal to 1 minus the correlation coefficient. The hierarchical clustering method was applied to the dissimilarity matrix and the resulting dendrogram of peptide clusters was presented.

Statistical significance was defined as *P* < 0.05. All statistics were calculated using R version 3.5.1 (R Foundation for Statistical Computing, Vienna, Austria). No adjustment was made for multiple comparisons.

### Data availability.

MRM-MS data are publicly available through Panorama Web (https://panoramaweb.org/project/Colorado%20State%20U%20-%20Dobos%20Lab/S26%20LTBI/begin.view).

## RESULTS

### Participants.

The majority of the 74 participants with LTBI were white (*n* = 44, 60%); 25 (34%) were black, 3 (4%) were Asian/Pacific Islander, one individual identified as American Indian/Alaskan Native (1.35%), and one individual as other (1.35%) (Table 2).

**TABLE 2:** Summary statistics of attributes measured in individuals with LTBI

Variable^*a*^	Female, *n* = 33	Male, *n* = 41
Median age (IQR)	36, (26–46)	41 (30–49)
Median BMI (IQR)	30 (25–35)	28 (24–33)
Race		
White	24 (73%)	20 (49%)
Black	7 (21%)	18 (44%)
Asian/Pacific Islander	2 (6%)	1 (2%)
American Indian/ Alaska Native	0 (0%)	1 (2%)
Other	0 (0%)	1 (2%)
History of alcohol use/abuse	21 (64%)	27 (66%)
Hepatitis comorbidity present	1 (3%)	4 (10%)
Close contact with TB patient	17 (52%)	26 (63%)
TST conversion	16 (49%)	15 (37%)
History of drug use	1 (3%)	4 (10%)
Unknown HIV status	6 (18%)	10 (24%)
Homeless	1 (3%)	7 (17%)
WHO birthplace outside of the Americas	2 (6%)	3 (7%)
History of smoking in past 5 years (not including current smokers)	2 (6%)	3 (7%)
Current smoker	10 (30%)	16 (39%)

a BMI, body mass index; IQR, interquartile range; TST, tuberculin skin test.

The study cohort was 45% female and 55% male; however, sex was not equally represented in the two major races (Table 2). Of the study participants, 41 (55.4%) were selected for LTBI treatment because they had LTBI and were close contacts of a TB case, while 31 (44.6%) had a documented conversion of the TST (Table 2). The majority of the participants had a history of alcohol use (*n* = 48, 64.9%), but only a few had a history of drug use (Table 2). Additional demographic information can be found in Table 2. Patient demographics did not associate with the MRM-MS results.

### Detection of M. tuberculosis peptides in serum EVs from individuals with LTBI.

**FIG 1:**
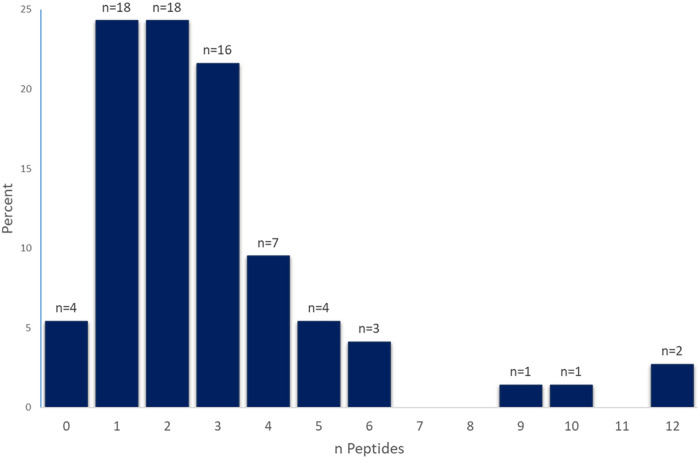
Number of peptides detected in individuals with LTBI. The *y* axis displays the percentage of individuals with LTBI; the *x* axis shows the number (0 to 12) detected peptides in individuals (*n*).

Samples from healthy controls were used to establish a cutoff value for confident detection of M. tuberculosis peptides (Supplemental Material 2). The majority of individuals with LTBI had at least one, two, or three peptides detected above the established cutoff value (24%, 24%, and 22%, respectively). Nearly one quarter (24%) of the participants had 4 or more peptides detected, with the maximum number of peptides (i.e., 12) identified in two individuals (Fig. 1). Only 4 (5%) individuals with LTBI did not have any detectable peptides. No demographic trait had a statistically significant association with number of peptides in a linear model (data not shown).

### Correlation analysis identified three clusters of peptides.

**FIG 2:**
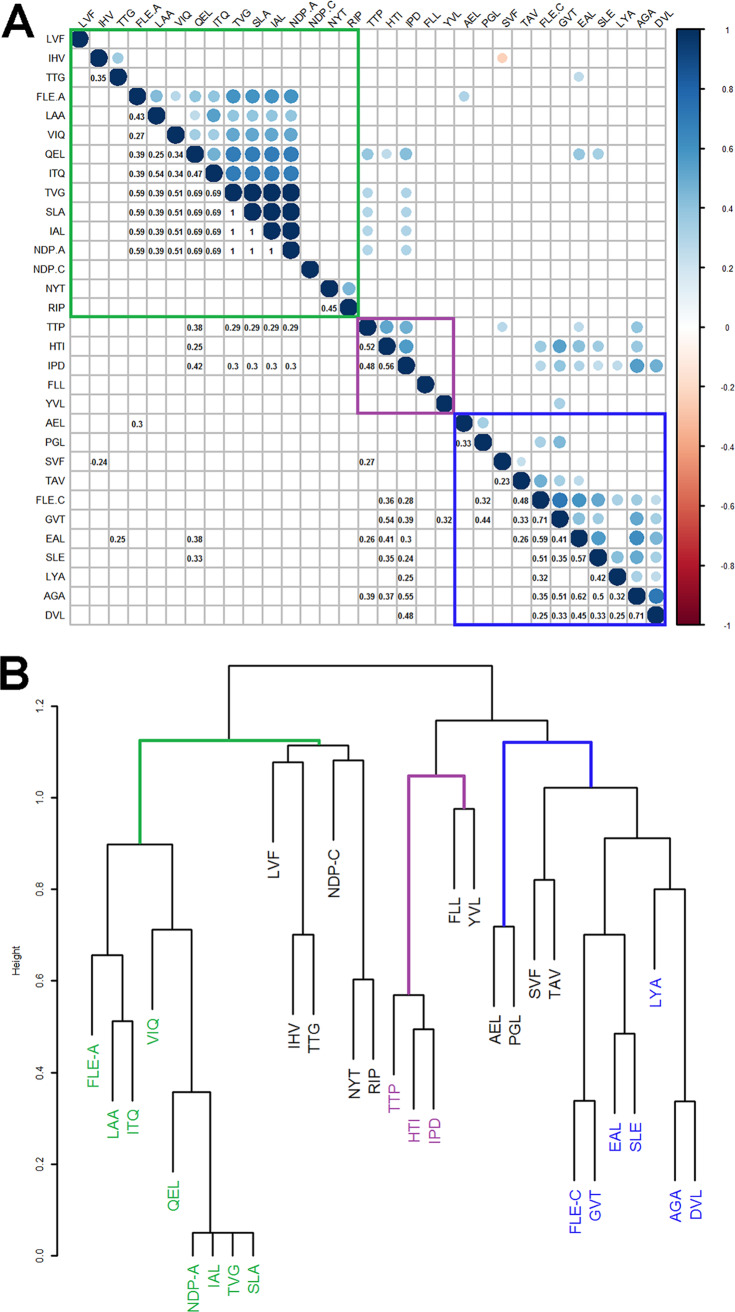
Results of cluster analysis of peptide concentrations. The three clusters identified are cluster 1 (green), cluster 2 (purple), and cluster 3 (blue). (A) Correlogram showing Spearman correlation coefficients (R) where *P* < 0.05. Upper circles are blue to indicate positive and orange to indicate negative R, and area of the circle corresponds with R absolute value; lower circles show corresponding numeric R. (B) Dendrogram showing the results of hierarchical clustering of a distance matrix calculated as dissimilarity = 1 – R.

Four peptides (IAL, NDP-A, SLA, and TVG) showed perfect correlation among one another as they were detected in only one individual with LTBI (coefficient: 1.0, *P* < 0.001; Fig. 2A). Most peptides had positive or no correlation with one another; IHV and SVF were the only peptides to have a significant inverse relationship as shown by a negative correlation coefficient (coefficient: –0.25, *P* = 0.04; Fig. 2A).

Peptides in the Spearman correlation matrix were ordered by hierarchical clustering to look for patterns. Three clusters were identified as follows: (i) cluster 1: FLE-A, LAA, ITQ, VIQ, QEL NDP-A, IAL, TVG, and SLA (Fig. 2A; green square); (ii) cluster 2: TTP, HTI, and IPD (Fig. 2A; purple square); and (iii) cluster 3: FLE-C, GVT, EAL, SLE, LYA, AGA, SVF, PGL, TAV and DVL (Fig. 2A; blue square). These clusters were also observed when we calculated a distance matrix (where dissimilarity was equal to 1 minus the correlation coefficient) and derived a dendrogram of clustered peptides (Fig. 2B).

### M. tuberculosis peptides detected in individuals with LTBI are involved in glutamine metabolism (GlnA and GarA), protein stability (GroES, DnaK), and fatty acid synthesis (AcpM).

**FIG 3:**
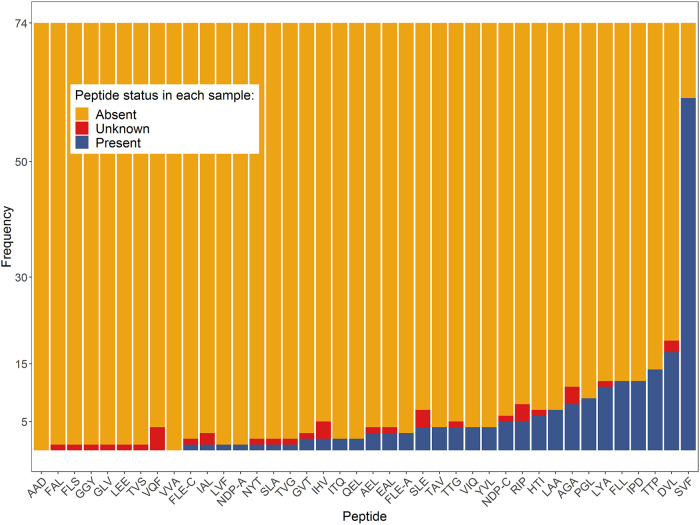
Distribution of absent, unknown, and detected peptides in the 74 study participants with LTBI. The *y* axis represents the frequency in which each individual peptide was detected. Orange represents absent (undetected) peptides, blue represents present (detected peptides), and red represents unknowns, i.e., peptides in which a measurement was not possible due to matrix effects.

Thirty-one of the forty evaluated peptides were detected in at least one subject (Table 3, Fig. 3). Peptides belonging to GlnA (SVF), GroES (DVL), DnaK (TTP), AcpM (IPD), and GarA (FLL) were the peptides most commonly detected, with all of them identified in at least 12 different individuals with LTBI (Table 3). Peptide SVF from GlnA1 was the most commonly identified peptide, with detection in 61 of the 74 participants (82%). While the remaining top 5 peptides were not identified nearly as frequently as SVF, they were detected in 17 (DVL), 14 (TTP), 12 (FLL), and 12 (IPD) of the individuals with LTBI. All participants in which peptide IPD (AcpM) was identified also had either TTP (DnaK) or DVL (GroES) detected in their serum EVs. When DVL, TTP, and FLL were combined, these peptides accounted for detection in 30 of the 74 participants (41%).

**TABLE 3:** Peptides detected in individuals with LTBI

Protein	Peptide	No. in which detected	Protein	Peptide	No. in which detected
**GlnA**	SVF	61			
**GroES**	DVL	17	**SahH**	GVT	2
	RIP	5		IHV	2
**DnaK**	TTP	14	**HspX**^*a*^	AEL	3
	ITQ	2	**BfrB**	EAL	3
**AcpM**^*a*^	IPD	12		AGA	8
	TVG	1	**MrsA**	TAV	4
**GarA**	FLL	12		YVL	4
	LVF	1	**Mpt64**	SLE	4
**Ag85A**	NDP-A	1	**Mpt32**	TTG	4
	FLE-A	3		LYA	11
**PpiA**	IAL	1	**GlcB**^*a*^	NYT	1
	VIQ	4	**Cfp2**^*a*^	SLA	1
	HTI	6	**Esat6**^*a*^	LAA	7
**Ag85C**^*a*^	FLE-C	1	**Ag85B**	PGL	9
	NDP-C	5			
**Cfp10**	QEL	2			

a Protein with additional monitored peptides. Additional peptides resulted in either unknowns or negatives. The five peptides highlighted in the text are: SVF, FLL, IPD, TTP and DVL.

### Proteins involved in nitrogen metabolism are among the top five proteins identified in EVs of individuals with LTBI.

Two of the five peptides most frequently detected in individuals with LTBI belonged to proteins GlnA1 and GarA, both of which are related to nitrogen metabolism by regulating the ratio of glutamate/glutamine in the cell. The peptide identified in the largest number of individuals with LTBI was SVF (*n* = 61, 82.4%), which belongs to the glutamine synthetase GlnA1 (Rv2220). We also observed GarA (Rv1827) as one of the most frequently detected proteins, with peptide FLL identified in 12 of the individuals with LTBI (16.2%).

### Two heat shock chaperonins are among the top five proteins detected in LTBI serum exosomes.

Along with GlnA1 and GarA, GroES and DnaK were among the five most commonly detected proteins in individuals with LTBI. Peptides DVL and RIP from GroES were identified in 17 (22.97%) and 5 (6.76%) of the LTBI participants, respectively, while peptides TTP and ITQ of DnaK were identified in 14 (18.92%) and 2 (2.70%) LTBI participants, respectively (Table 3, Fig. 3). DnaK (Rv0350) is an essential chaperone (37–39), and together with GroES (among others) was identified in immunodominant fractions with T-cell activity (40), corroborating early observations of M. tuberculosis antigens that elicit T-cell responses (41).

## DISCUSSION

In this study, we demonstrate that in our tested cohort at least one M. tuberculosis peptide was identified in the serum EVs of 95% of persons infected with M. tuberculosis (Fig. 1). This result was achieved by applying an optimized data analysis that accounts for false-positive signals (present in complex matrices) and calculating a threshold of positivity for each peptide to increase the confidence of peptide identification in the LTBI samples (see Supplemental Material 2).

Of the 40 monitored peptides, a single peptide from GlnA1 was identified in 82% of the study participants. In addition to GlnA1, other proteins, including GarA, the chaperones GroES and DnaK, and the fatty acid synthase AcpM, were identified in at least 16% of the LTBI participants. All of these proteins have been shown to have some role during dormancy or key physiological processes of M. tuberculosis (42–47), and our findings indicate they may be good candidates to be considered biomarkers of LTBI.

GlnA1 and GarA are involved in nitrogen metabolism (see [48] for review). GlnA1 is essential for both *in vitro* (48) and *in vivo* growth (49). It is involved in the synthesis of glutamine from l-glutamate and ammonium, serving not only as a means for nitrogen assimilation (50), but also as a building block for the synthesis of cell wall poly-α-l-glutamine (43, 51, 52). GlnA1 activity is regulated by adenylation via GlnE, which senses the ratio of 2-oxoglutarate (2-OG) and glutamine, two products that are directly related to availability of nitrogen in the cell (42, 45). Likewise, GarA, named as glycogen accumulation regulator, is involved in glutamate storage and metabolism by regulating the interconversion of glutamate and 2-OG (45, 53). GarA inhibits the activities of glutamate dehydrogenase (Gdh) and ketoglutarate decarboxylase (KGD) while stimulating the activity of the glutamine oxoglutarate aminotransferases (GltB and GltD), effectively promoting glutamate synthesis and decreasing entry of 2-OG into the tricarboxylic acid (TCA) cycle. While the exact role of GlnA1 and GarA in nitrogen metabolism during *in vivo* conditions has not been explored, some studies point to the relevance of these two proteins during infection. For instance, GlnA1 has been shown to be involved in cell wall resistance and pathogenicity in M. bovis (43), and phosphorylation of GarA by PknG has been implicated in increasing survival of latent M. tuberculosis (54).

At the host level, GlnA1 induces cellular immune responses and has been proposed as a novel vaccine candidate component (44). Even though GlnA1 is not an actively secreted protein, its high extracellular stability, as well as high expression (48), result in large quantities being found in the extracellular milieu of *in vitro* cultures (55). On the contrary, when M. tuberculosis is grown in pellicle form, GlnA1 is found to be highly abundant in the bacterial membrane compared to shaken cultures, suggesting that expression and/or localization of GlnA1 may be dependent on growth conditions (56). This is relevant to both host exosome localization, as well as its significance in LTBI. For instance, biofilm growth (such as growth in surface pellicles) has been implicated as a possible source of aerosol transmission during TB disease (56), but it has also been proposed as a mechanism of persistence during dormancy (57). It is possible that higher expression of GlnA1 during LTBI may be a result of biofilm-related M. tuberculosis persistence, and thus why its peptide is readily detected in LTBI and less so in active TB. Although M. tuberculosis proteins circulating in exosomes arise mostly from intracellular infection, phagocytosis of extracellular debris (such as released proteins from biofilm formation) can also be a source of exosome content (30). Additional studies are needed in order to determine if GlnA1 and/or GarA can be exploited as biomarkers of LTBI, as well as their potential role (if any) during LTBI. Our preliminary analysis comparing our results for GlnA1 (SVF peptide) to a subset of active TB patients from our previous study (25) indicates this peptide may be an LTBI biomarker candidate for further validation studies (Supplemental Material 3).

GroES and DnaK are both chaperones involved in the cellular response to stresses and AcpM is an acyl carrier protein involved in mycolic acid biosynthesis, a key step in the synthesis of cell walls (46, 48, 55, 58). The function of GroES (Rv3418c) is annotated as a suppressor of the ATPase activity of GroEL1, another chaperonin that prevents misfolding of proteins and promotes proper protein folding during stress conditions. GroES is also referred to as the 10-kDa antigen because of its ability to elicit T-cell responses (59, 60). GroES appears to be highly abundant in the cytosol of M. tuberculosis grown under anaerobic conditions (61), but is also one of the most abundant proteins under regular growth conditions (62). GroES was initially identified in human serum exosomes of individuals with TB infection, although its presence was only observed in a few individuals with pulmonary and extra pulmonary TB (24). However, GroES association with EVs in the context of M. tuberculosis infection was later confirmed in the guinea pig model, in which GroES was identified in urine, serum, and bronchoalveolar lavage EVs of guinea pigs infected with M. tuberculosis (63). GroES was also demonstrated to be ubiquitinated as a requirement for exosome transport (64).

GroES and AcpM are also among the most abundant proteins produced by M. tuberculosis both during exponential growth as well as during dormancy (62). It is thus possible that the success of detecting these proteins in host EVs may be due to their high expression during *in vivo* infection. While this hypothesis remains to be tested, there is evidence that these antigens are presented by the host during infection. GroES, DnaK, and AcpM have been shown to elicit IFN-γ responses in individuals with LTBI. These proteins elicited IFN-γ in more than 80% of household contacts, but in only 58%, 23%, and 45% of pulmonary TB patients, respectively, indicating that these antigens may be more readily recognized by the host during LTBI (65).

The set of proteins identified in serum EVs from study participants with LTBI differ from those previously identified in patients with TB disease, despite that the same MRM and processing methods were used (25). The most commonly detected peptides in TB patients in the study by Mehaffy et al. belonged to proteins Cfp2 and Mpt32, while peptides from GlnA, GroES, DnaK, AcpM, and GarA were only detected in a small number of the study participants (25). Indeed, the SVF peptide, found in the most persons in this LTBI study, was rarely detected in TB patients (Supplemental Material 3). Peptides most frequently detected in the current study also differ from our initial exploratory study which included individuals with TB and with LTBI (24). For instance, AcpM, GroES, and DnaK were among the proteins which our group originally identified as discriminatory of TB versus LTBI or uninfected hospitalized patients (24). It is possible that these differences may be due to our improved methodologies (including the use of synthetic internal standards), different cohort, and/or demographic factors such as birthplace or geographical location, rather than TB versus LTBI. However, it is also plausible that the physiologic state of M. tuberculosis varies across the spectrum of latency to clinical disease, and the differences in proteins are a reflection of this variability. Our traditional definitions of LTBI also likely represent a dynamic spectrum with different stages, from recent infection to reactivation leading to TB disease (66–75). It is possible, then, that M. tuberculosis proteins such as GlnA, GroES, DnaK, and GarA may fluctuate in abundance between physiologic states, and thus could be evaluated as prognostic reagents for detection of progression from asymptomatic infection to disease.

Our study has limitations. First, the MRM methodology used in the present study was initially developed using a set of proteins optimized for the detection in serum EVs in TB patients (25). The observations from this study, namely, that M. tuberculosis peptides are abundantly detected in LTBI samples, along with further support for the potential for different peptide profiles to distinguish between LTBI and TB, was unexpected. Thus, we expect that additional proteins/peptides may be discovered specifically in individuals with LTBI. Additionally, our study was limited to a qualitative analysis (presence versus absence) of M. tuberculosis peptides. Future studies will focus on assessing whether quantitative differences in peptide abundance also provide further insight when comparing TB versus LTBI, or for LTBI before and after treatment. Lastly, our analyses are based on convenience samples collected as part of a clinical trial, and as such provide exploratory evidence of M. tuberculosis peptide detection in LTBI samples, which needs to be confirmed in samples collected from well-characterized diagnostic cohorts.

**Conclusion.** We have shown that M. tuberculosis peptides can be detected in serum extracellular vesicles of individuals with LTBI. Furthermore, one of the peptides (SVF from GlnA1) was found in 82% of study participants. While our findings need to be validated in a larger and more geographically diverse cohort, the peptide SVF, along with other peptides detected here, may be good candidates for the development of point of care (POC) tests for the diagnosis of LTBI.

## Supplementary Material

Supplemental file 1

Supplemental file 2

Supplemental file 3
