# Supplementary material for: Identification of Mycobacterium tuberculosis Peptides in Serum Extracellular Vesicles from Persons with Latent Tuberculosis Infection
Source: J Clin Microbiol. 2020 May 26;58(6):e00393-20. doi: 10.1128/JCM.00393-20 (PMC7269374; doi:10.1128/JCM.00393-20)
Supplement: Supplemental file 1 [file JCM.00393-20-s0001.pdf]

```
/**********************************************************************************************************/
/*              Program Name:           Skyline_Processing.sas                                                    */
/*              Program Written by:     Charis Willyerd & Nicole Kruh-Garcia          April 15, 2018             */
/**********************************************************************************************************/

/*Create library where CSV/Excel/SAS files are and will get output*/

/*libname folder 'C:\Users\diape\Desktop\Folder SAS Stuff\Folder SAS\SAS libname';*/
/*folder = where the datasets are located on your PC, change this to your local directory*/

libname folder 'c:\users\folder\documents\my_sas_files\folder';

/*This should be the same location as folder, but without the quotes*/
%let infile = c:\users\folder\documents\my_sas_files\folder;

/*Import datasets --> This macro will import a csv file*/
%macro import (skylineinfile,sasfile);
proc import out         =       folder.&sasfile.
            datafile    =       "&infile.\SkylineOutput.csv."
            dbms        =       csv replace;
            getnames    =       yes;
            datarow             = 2;
            guessingrows=       max;
/*guessingrows = 1000 means SAS looks at 1000 rows to determine the length of a column. This only works for CSV files, not Excel*/
run;
%mend import;

/*Import SkylineOutput*/
%import(skyline, SkylineOutput);

/***************************************************************************************************************\

Purpose of Program:

For each peptide/standard pair (represented once in each patient sample):
            Ex: Peptides Sequence (column name): AELPGVDPDK
            Ex: Replicate (column name): patient sample number

Align all transition areas for native (light) peak (3 to 5 columns)
            Ex: y8, y7, y5, y4 for Precursor Mz: 520.7666 (each y ion also has an associated Product Mz)

Align all transition areas for standard (heavy) peak (same number of columns as native)
            Ex: y8, y7, y5, y4 for Precursor Mz: 524.7737

Convert all #n/a to zero

Divide native peak/standard peak for each transition
            Ex: y8 native/ y8 standard

Sum all peak/standard areas (this value = normalized total peak area or nTPA)

If all standard peak values = 0, then designate the nTPA as -1

If less than three non-zero values in native peak, then designate the nTPA as 0 (unless designated -1)

Create delta DotP value: Library dot Product (column name) for native - library dot product for standard; absolute value

If the delta DotP value is equal to or less than .15 report nTPA; if below report 0 (unless -1) (output 1)

If the delta DotP value is equal to or less than .1 report nTPA; if below report 0 (unless -1) (output 2)

If the delta DotP value is equal to or less than .15 report TAR; if below report 0 (unless -1) (output 3)

If the delta DotP value is equal to or less than .1 report TAR; if below report 0 (unless -1) (output 4)

The TAR is only present in native peak rows (only one value per patient sample)
```

Link in a healthy threshold file (use peptide name and a value associated with it in a second column). If no file associated, leave outputs 5-8 as null.

If output 1 = above healthy threshold for peptide then report nTPA; if below report 0 (unless -1) (output 5)

If output 2 = above healthy threshold for peptide then report nTPA; if below report 0 (unless -1) (output 6)

If output 3 = above healthy threshold for peptide then report TAR; if below report 0 (unless -1) (output 7)

If output 4 = above healthy threshold for peptide then report TAR; if below report 0 (unless -1) (output 8)

```
\*********************************************************************************************************************/


/*        START CODE        */

options
errors = 5
mprint
mfile
compress=yes;

/*Convert data into correct type (character or numeric) and deal with nulls and zeros*/
data folder.nulls;
        set folder. SkylineOutput;

        /*Convert all #n/a to zero --> code only converts these two columns. Change Total_Area_Ratio & Library_Dot_Product from
        character to numeric type data*/
        if total_area_ratio = '#N/A' then total_area_ratio = '0';

        if library_dot_product = '#N/A' then library_dot_product = '0';

        if area = '#N/A' then area = '0';

        /*Need a unique ID (aka: primary key) to be able to separate out native and standard peaks. Concatenate vars to create a primary key*/
        /*peprep was initiated in folder code as PepSeqRep*/
        length unique_ID                $50.        ;
                Unique_ID = compress(replicate_name||"_"||peptide_sequence||"_"||fragment_ion);
                PepSeqRep = compress(peptide_sequence||"_"||replicate_name);

        /*Convert total area ratio to numeric - calculations won't work if character*/
        total_area_ratio_new = input(total_area_ratio, BEST12.);

        library_dot_product_new = input(library_dot_product, BEST12.);

        area_new = input(area, BEST12.);

        /*Drop old character value total_area_ratio and library_dot_product columns. Replace with newly created numeric columns
        (library_dot_product_new and total_area_ratio_new), but using the original column name*/

        drop total_area_ratio library_dot_product area;

        rename total_area_ratio_new = total_area_ratio;
        rename library_dot_product_new = library_dot_product;
        rename area_new = area;

run;

/*Sort Native_Precursor_Area and Isotopic_Precursor_Area into separate datasets accordingly. Rename the var "area"*/
proc sort data=folder.nulls;
        by unique_ID PepSeqRep precursor_mz;
run;

data folder.isotopic folder.native;
        set folder.nulls;
                by unique_id;
                if first.unique_id then do;
                        native_precursor_area = area;
                end;
                if last.unique_id then do;
```

```
                isotopic_precursor_area = area;
        end;
run;

/*Split native data from isotopic. Delete null native_precursor_area. Rename vars.*/
proc sort data=folder.native;
    by native_precursor_area ;
run;

data folder.native_only;
    set folder.native;
    if native_precursor_area = "" then delete;
        rename library_dot_product = native_dotP;
        rename precursor_mz = native_precursor_Mz;
        rename product_mz = native_product_Mz;
        drop area isotopic_precursor_area;
run;

proc sort data=folder.native_only;
    by PepSeqRep Fragment_ion;
run;

proc sort data=folder.isotopic;
    by isotopic_precursor_area ;
run;

data folder.isotopic_only;
    set folder.isotopic;
    if isotopic_precursor_area = "" then delete;
        rename library_dot_product = isotopic_DotP;
        rename precursor_mz = isotopic_precursor_Mz;
        rename product_mz = isotopic_product_Mz;
        drop area native_precursor_area total_area_ratio;
run;

proc sort data=folder.isotopic_only;
    by PepSeqRep Fragment_ion;
run;

/*Left join folder.isotopic_only and folder.native_only on unique_id*/
proc sql;
    create table
        folder.combined             as
    select
        n.peptide_sequence          ,
        n.protein_name              ,
        n.replicate_name            ,
        n.fragment_ion              ,
        i.isotopic_precursor_mz     ,
        i.isotopic_product_mz       ,
        i.isotopic_precursor_area   ,
        i.isotopic_Dotp             ,
        i.unique_id                 ,
        i.PepSeqRep                 ,
        n.native_precursor_mz       ,
        n.native_product_mz         ,
        n.native_precursor_area     ,
        n.native_dotp               ,
        n.total_area_ratio
    from
        folder.isotopic_only        as i
    left join
        folder.native_only          as n
    on
        i.unique_id                 =
        n.unique_id
    ;
quit;
```

```
data folder.combined2;
		set folder.combined;

		length		ratio delta_dotp 8
					pep_rep $50.;

		/*Divide native_precursor_area by isotopic_precursor_area to create new var ratio*/
		ratio = native_precursor_area / isotopic_precursor_area;

		/*Subtract native_dotp from isotopic_dotp, using absolute values to get delta_DotP*/
		delta_DotP = abs(isotopic_dotp - native_dotp);

		/*Concatenate peptide_sequence and replicate_name to get new var pep_rep*/
		pep_rep = compress(peptide_sequence||trim(replicate_name));

		format ratio z6.4;
		drop pep_rep;

run;

proc sort data=folder.combined2;
		by PepSeqRep fragment_ion;
run;

/*Not true array. Create new var vatio_y# from fragment_ion value*/
data folder.combinedarray;
		set folder.combined2;

				if fragment_ion = 'y3' then do;
						ratio_y3 = ratio;
				end;

				if fragment_ion = 'y4' then do;
						ratio_y4 = ratio;
				end;

				if fragment_ion = 'y5' then do;
						ratio_y5 = ratio;
				end;

				if fragment_ion = 'y6' then do;
						ratio_y6 = ratio;
				end;

				if fragment_ion = 'y7' then do;
						ratio_y7 = ratio;
				end;

				if fragment_ion = 'y8' then do;
						ratio_y8 = ratio;
				end;

				if fragment_ion = 'y9' then do;
						ratio_y9 = ratio;
				end;

				if fragment_ion = 'y10' then do;
						ratio_y10 = ratio;
				end;

				if fragment_ion = 'y11' then do;
						ratio_y11 = ratio;
				end;

				if fragment_ion = 'y12' then do;
						ratio_y12 = ratio;
				end;

				if fragment_ion = 'y13' then do;
						ratio_y13 = ratio;
```

```
		end;

		if fragment_ion = 'y14' then do;
			ratio_y14 = ratio;
		end;

		if fragment_ion = 'y15' then do;
			ratio_y15 = ratio;
		end;

		if fragment_ion = 'y16' then do;
			ratio_y16 = ratio;
		end;

		if fragment_ion = 'y17' then do;
			ratio_y17 = ratio;
		end;

		if fragment_ion = 'y18' then do;
			ratio_y18 = ratio;
		end;

		if fragment_ion = 'y19' then do;
			ratio_y19 = ratio;
		end;

		if fragment_ion = 'y20' then do;
			ratio_y20 = ratio;
		end;

		if fragment_ion = 'y21' then do;
			ratio_y21 = ratio;
		end;

		if fragment_ion = 'y22' then do;
			ratio_y22 = ratio;
		end;

		if fragment_ion = 'y23' then do;
			ratio_y23 = ratio;
		end;

		if fragment_ion = 'y24' then do;
			ratio_y24 = ratio;
		end;

		if fragment_ion = 'y25' then do;
			ratio_y25 = ratio;
		end;
run;

data folder.combinedarray2;
	set folder.combinedarray;

		drop
			fragment_ion
			unique_id
			isotopic_precursor_mz
			native_precursor_mz
			isotopic_product_mz
			native_product_mz
			isotopic_dotp
			native_dotp
			ratio;

run;

proc sort data=folder.combined2;
	by PepSeqrep;
run;
```

```
/*Transpose data*/
proc transpose data=folder.combined2 out=folder.combinedtransposed (drop=_NAME_ ) prefix=ratio;
	var ratio;
	by PepSeqrep;
run;

proc transpose data=folder.combined2 out=folder.combinedtransposed2 (drop=_NAME_ ) prefix=fragment_ion;
	var fragment_ion;
	by PepSeqrep;
run;

/*Inner join on PepSeqrep*/
proc sql;
	create table
		folder.combinedtransposed3	as
	select
		one.*			,
		two.*
	from
		folder.combinedtransposed	as one
	inner join
		folder.combinedtransposed2	as two
	on
		one.PepSeqrep		=
		two.PepSeqrep
;
quit;

/*Inner join on PepSeqrep*/
proc sql;
	create table
		folder.combine_array_transposed		as
	select
		one.*			,
		two.*
	from
			folder.combinedarray2		as one
	inner join
			folder.combinedtransposed3	as two
	on
		one.PepSeqrep		=
		two.PepSeqrep
	;
quit;

data folder.combine_array_transposed2;
	set folder.combine_array_transposed;
	drop ratio_y3-ratio_y25;
run;

/*Sum ratio1 – ratio 5 to create new va nTPA*/
data folder.sum;
	set folder.combine_array_transposed2;
		nTPA = sum(of ratio1 ratio2 ratio3 ratio4 ratio5);
run;

proc sort data=folder.sum;
	by PepSeqRep;
run;

/*Convert 0's to -1 for isotopic_precursor_area*/
data folder.counting;
	set folder.sum;
		by PepSeqRep;

			length native_counter 8;

			/*convert ipa = 0 to -1*/
			if isotopic_precursor_area = 0 then do;
				indeterminate_counter = -1;
```

```
                        end;
                        if isotopic_precursor_area > 1 then do;
                                indeterminate_counter = 0;
                        end;

                        /*if >3 non-zero values in native peak per peprep, ntpa = 0 unless -1*/
                        if native_precursor_area >0 then do;
                                native_counter = 1;
                        end;
                        if native_precursor_area ^ >0 then do;
                                native_counter = 0;
                        end;

run;


/*Count number of indeterminates per pep_rep then make rule for counting*/
proc sql;
        create table
                folder.counting2                as
        select
                *                                               ,
                sum(native_counter)             as nativecounter_sum ,
                sum(indeterminate_counter)      as indeterminate_sum
        from
                folder.counting
        group by
                PepSeqRep
        order by
                PepSeqRep
        ;
quit;

/*Create nativecounter_sum and indeterminate_sum*/
proc sql;
        create table
                folder.counting3
        as
        select
                *                                                                               ,
                (case when nativecounter_sum <3         then 0  else . end)     as ntpa_final           ,
                (case when indeterminate_sum <-2        then -1 else . end)     as indeterminate_final
        from
                folder.counting2
        group by
                PepSeqRep
        order by
                PepSeqRep
        ;
quit;

/* If first.PepSeqRep then create new var PepSeqRep using value from first_PepSeqRep*/
data folder.count_collapsed;
        set folder.counting3;
                by PepSeqRep;
                        if first.PepSeqRep then do;
                                first_PepSeqRep = PepSeqRep;
                        end;
run;

proc sort data=folder.count_collapsed;
        by first_PepSeqRep ;
run;

/*Delete null first_PepSeqRep*/
data folder.count_collapsed2;
        set folder.count_collapsed;
        if first_PepSeqRep = "" then delete;
run;
```

```
/*Drop unnecessary vars & cleanup datasets*/
data folder.count_collapsed3;
		set folder.count_collapsed2;
		drop
				native_counter
				indeterminate_counter
				nativecounter_sum
				indeterminate_sum
				first_pepseqrep
				isotopic_precursor_area
				native_precursor_area;
		run;

data folder.reordered;
		retain protein_name peptide_sequence replicate_name total_area_ratio indeterminate_final ntpa_final delta_dotp ntpa;
		set folder.count_collapsed3;
run;

proc sort data=folder.reordered;
		by peptide_sequence replicate_name ;
run;

/* 0 is the result if not -1 and present in ntpa_final */

data folder.Final_nTPA;
		set folder.reordered;
		if indeterminate_final -1 then Final_nTPA = -1;
		else if ntpa_final = 0 then Final_nTPA = 0;
		else Final_nTPA = ntpa;
run;

/* 0 is the result if not -1 and present in TAR_final */
data folder.Final_nTPA_TAR;
		set folder.Final_nTPA;
				if indeterminate_final -1 then Final_TAR = -1;
				else if ntpa_final = 0 then Final_TAR = 0;
				else Final_TAR = total_area_ratio;
run;

/*Final output rules */
data folder.Output1to4;
		set folder.Final_nTPA_TAR;
				if delta_dotp =< .15 then Output_1 = Final_nTPA;
				else if indeterminate_final = -1 then Output_1 = -1;
				else Output_1 = 0;

				if delta_dotp =< .1 then Output_2 = Final_nTPA;
				else if indeterminate_final = -1 then Output_2 = -1;
				else Output_2 = 0;

				if delta_dotp =< .15 then Output_3 = Final_TAR;
				else if indeterminate_final = -1 then Output_3 = -1;
				else Output_3 = 0;

				if delta_dotp =< .1 then Output_4 = Final_TAR;
				else if indeterminate_final = -1 then Output_4 = -1;
				else Output_4 = 0;
		run;

data folder.Results_Clean;
		set folder.Output1to4;
				drop
				total_area_ratio
				delta_dotp
				ntpa
				pepseqrep
				ratio1-ratio5
				fragment_ion1-fragment_ion5
				final_ntpa
				final_tar;
```

```
run;


/*make separate files for each peptide, so that when exported each can be added to Excel as a separate tab */

proc sort data= folder.Results_Clean;
		by peptide_sequence ;
run;

/*Create new datasets by peptide values*/
data folder.AAD_only;
		set folder.Results_Clean;
		if peptide_sequence ^= "AADMWGPSSDPAWER" then delete;
run;

data folder.AEL_only;
		set folder.Results_Clean;
		if peptide_sequence ^= "AELPGVDPDK" then delete;
run;

data folder.AGA_only;
		set folder.Results_Clean;
		if peptide_sequence ^= "AGANLFELENFVAR" then delete;
run;

data folder.DVL_only;
		set folder.Results_Clean;
		if peptide_sequence ^= "DVLAVVSK" then delete;
run;

data folder.EAL_only;
		set folder.Results_Clean;
		if peptide_sequence ^= "EALALALDQER" then delete;
run;

data folder.FAL_only;
		set folder.Results_Clean;
		if peptide_sequence ^= "FALNAANAR" then delete;
run;

data folder.FLE-A_only;
		set folder.Results_Clean;
		if peptide_sequence ^= "FLEGFVR" then delete;
run;

data folder.FLE-C_only;
		set folder.Results_Clean;
		if peptide_sequence ^= "FLEGLTLR" then delete;
run;

data folder.FLL_only;
		set folder.Results_Clean;
		if peptide_sequence ^= "FLLDQAITSAGR" then delete;
run;

data folder.FLS_only;
		set folder.Results_Clean;
		if peptide_sequence ^= "FLSAATSSTPR" then delete;
run;

data folder.GGY_only;
		set folder.Results_Clean;
		if peptide_sequence ^= "GGYFPVAPNDQYVDLR" then delete;
run;

data folder.GSL_only;
		set folder.Results_Clean;
		if peptide_sequence ^= "GSLVEGGIGGTEAR" then delete;
run;
```

```
data folder.GVT_only;
		set folder.Results_Clean;
		if peptide_sequence ^= "GVTEETTTGVLR" then delete;
run;

data folder.HTI_only;
		set folder.Results_Clean;
		if peptide_sequence ^= "HTIFGEVIDAESQR" then delete;
run;

data folder.IAL_only;
		set folder.Results_Clean;
		if peptide_sequence ^= "IALFGNHAPK" then delete;
run;

data folder.IHV_only;
		set folder.Results_Clean;
		if peptide_sequence ^= "IHVEALGGHLTK" then delete;
run;

data folder.IPD_only;
		set folder.Results_Clean;
		if peptide_sequence ^= "IPDEDLAGLR" then delete;
run;

data folder.ITQ_only;
		set folder.Results_Clean;
		if peptide_sequence ^= "ITQDLLDR" then delete;
run;

data folder.LAA_only;
		set folder.Results_Clean;
		if peptide_sequence ^= "LAAAWGGSGSEAYQGVQQK" then delete;
run;

data folder.LEE_only;
		set folder.Results_Clean;
		if peptide_sequence ^= "LEEENPEAAQALR" then delete;
run;

data folder.LVF_only;
		set folder.Results_Clean;
		if peptide_sequence ^= "LVFLTGPK" then delete;
run;

data folder.LYA_only;
		set folder.Results_Clean;
		if peptide_sequence ^= "LYASAEATDSK" then delete;
run;

data folder.NDP-A_only;
		set folder.Results_Clean;
		if peptide_sequence ^= "NDPLLNVGK" then delete;
run;

data folder.NDP-C_only;
		set folder.Results_Clean;
		if peptide_sequence ^= "NDPMVQIPR" then delete;
run;

data folder.NYT_only;
		set folder.Results_Clean;
		if peptide_sequence ^= "NYTAPGGGQFTLPGR" then delete;
run;

data folder.PGL_only;
		set folder.Results_Clean;
		if peptide_sequence ^= "PGLPVEYLQVPSPSMGR" then delete;
run;
```

```
data folder.QEL_only;
		set folder.Results_Clean;
		if peptide_sequence ^= "QELDEISTNIR" then delete;
run;

data folder.RIP_only;
		set folder.Results_Clean;
		if peptide_sequence ^= "RIPLDVAEGDTVIYSK" then delete;
run;

data folder.SLA_only;
		set folder.Results_Clean;
		if peptide_sequence ^= "SLADPNVSFANK" then delete;
run;

data folder.SLE_only;
		set folder.Results_Clean;
		if peptide_sequence ^= "SLENYIAQTR" then delete;
run;

data folder.SVF_only;
		set folder.Results_Clean;
		if peptide_sequence ^= "SVFDDGLAFDGSSIR" then delete;
run;

data folder.TAV_only;
		set folder.Results_Clean;
		if peptide_sequence ^= "TAVEQAAAELGDTGR" then delete;
run;

data folder.TTG_only;
		set folder.Results_Clean;
		if peptide_sequence ^= "TTGDPPFPGQPPPVANDTR" then delete;
run;

data folder.TTP_only;
		set folder.Results_Clean;
		if peptide_sequence ^= "TTPSIVAFAR" then delete;
run;

data folder.TVG_only;
		set folder.Results_Clean;
		if peptide_sequence ^= "TVGDVVAYIQK" then delete;
run;

data folder.SVF_only;
		set folder.Results_Clean;
		if peptide_sequence ^= "TVSLPVGADEDDIK" then delete;
run;

data folder.VIQ_only;
		set folder.Results_Clean;
		if peptide_sequence ^= "VIQGFMIQGGDPTGTGR" then delete;
run;

data folder.VQF_only;
		set folder.Results_Clean;
		if peptide_sequence ^= "VQFQGGGPHAVYLLDGLR" then delete;
run;

data folder.VVA_only;
		set folder.Results_Clean;
		if peptide_sequence ^= "VVADLTPQNQALLNAR" then delete;
run;

data folder.YVL_only;
		set folder.Results_Clean;
		if peptide_sequence ^= "YVLEELR" then delete;
run;
```
