# Supplementary material for: Identification of Mycobacterium tuberculosis Peptides in Serum Extracellular Vesicles from Persons with Latent Tuberculosis Infection
Source: J Clin Microbiol. 2020 May 26;58(6):e00393-20. doi: 10.1128/JCM.00393-20 (PMC7269374; doi:10.1128/JCM.00393-20)
Supplement: Supplemental file 2 [file JCM.00393-20-s0002.pdf]

Supplementary Data 2

Normalized Total Peak Area (nTPA) of healthy controls and individuals with LTBI for each assayed peptide. The dotted line corresponds to the calculated threshold of positivity (95 percentile of healthy samples). Graphs with no dotted line indicates a threshold = 0.

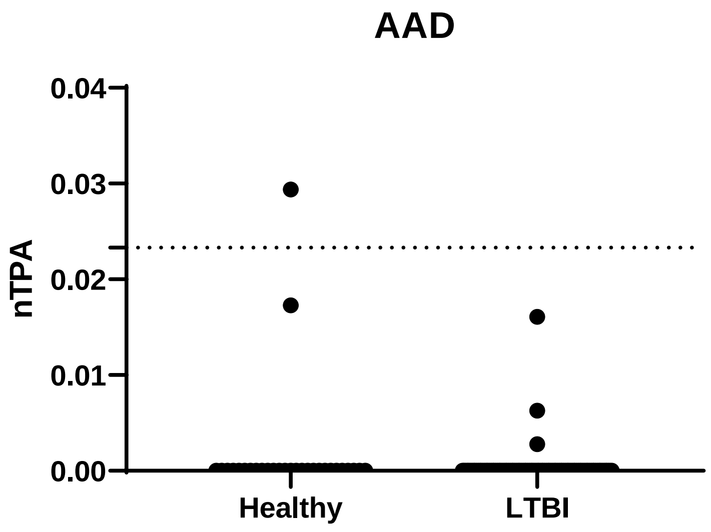

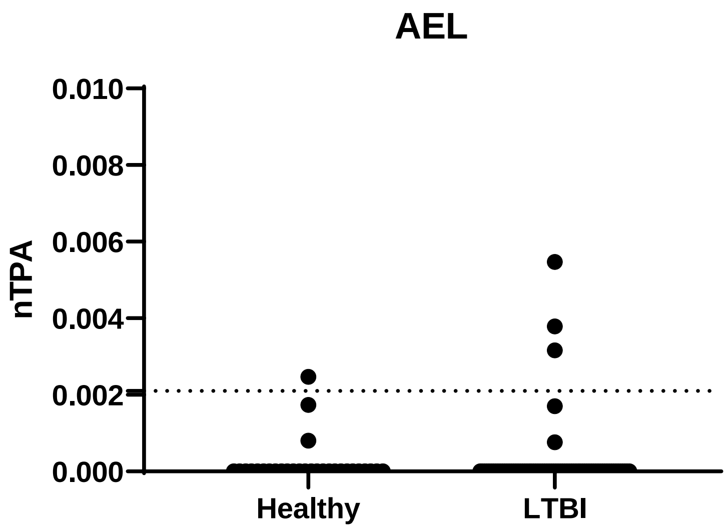

AGA

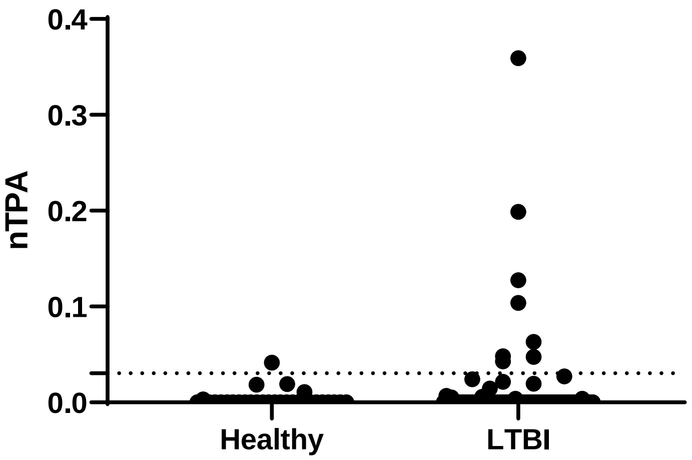

DVL

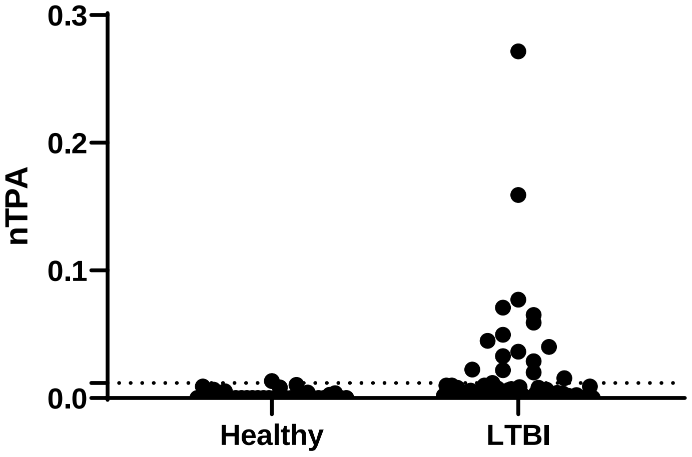

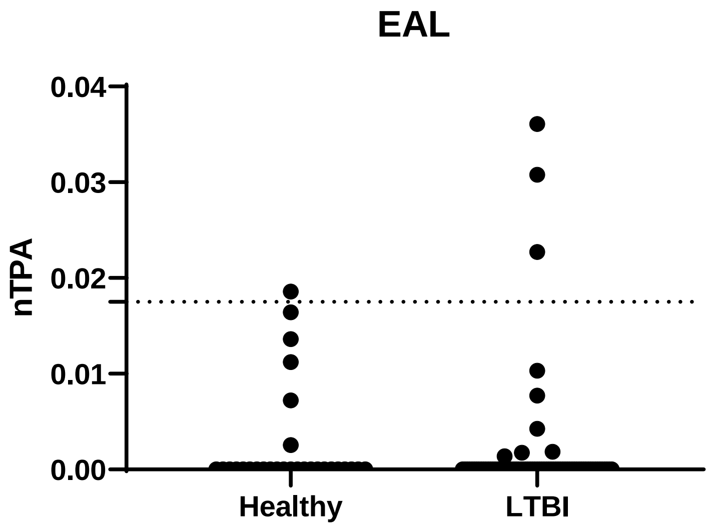
EAL
nTPA
0.04
0.03
0.02
0.01
0.00
Healthy
LTBI

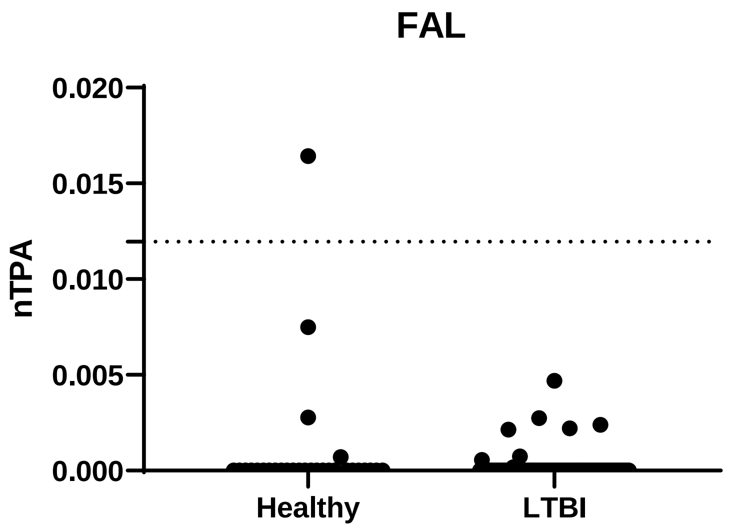
FAL
nTPA
0.020
0.015
0.010
0.005
0.000
Healthy
LTBI

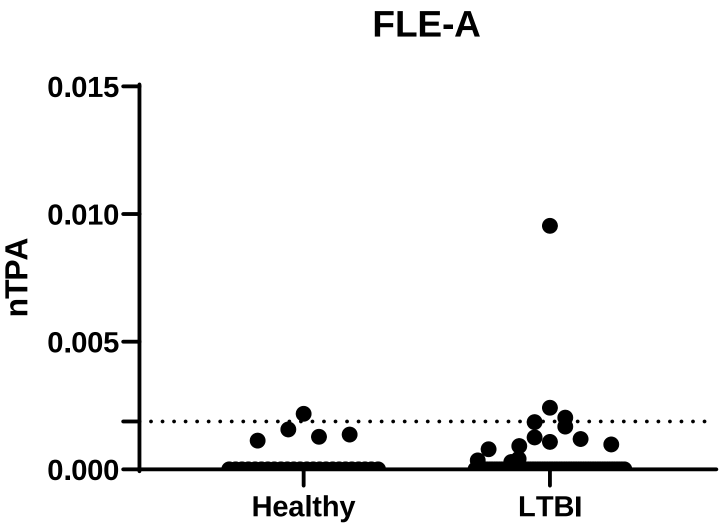
FLE-A
nTPA
0.015
0.010
0.005
0.000
Healthy
LTBI

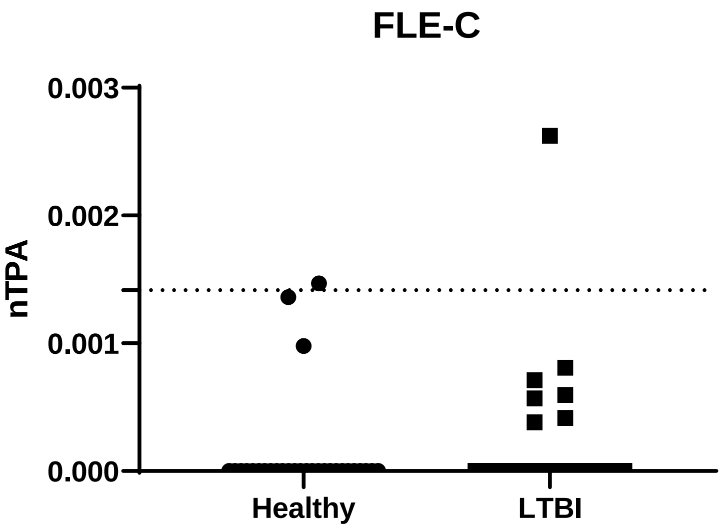
FLE-C
nTPA
0.003
0.002
0.001
0.000
Healthy
LTBI

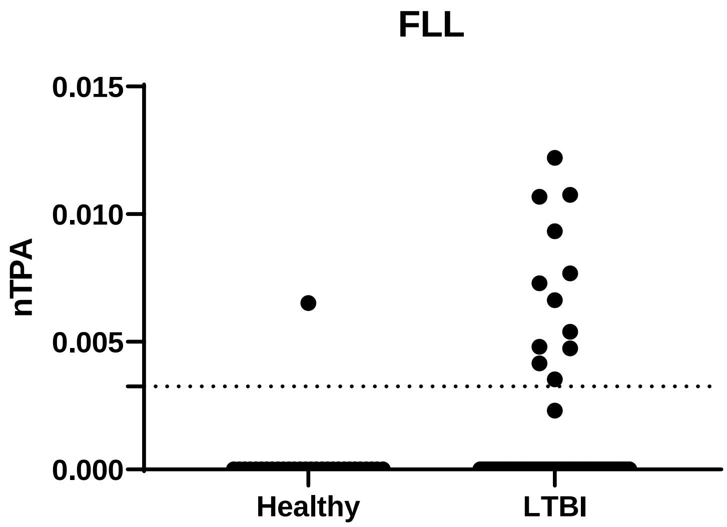
FLL
nTPA
0.015
0.010
0.005
0.000
Healthy
LTBI

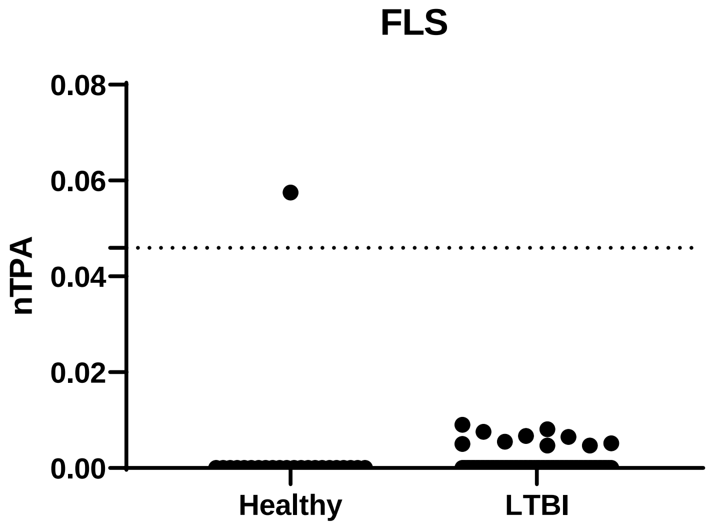
FLS
nTPA
0.08
0.06
0.04
0.02
0.00
Healthy
LTBI

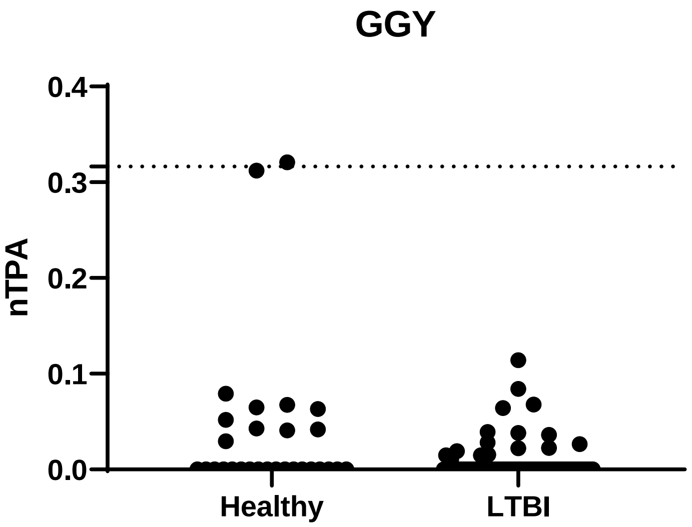
GGY
nTPA
0.4
0.3
0.2
0.1
0.0
Healthy
LTBI

sal

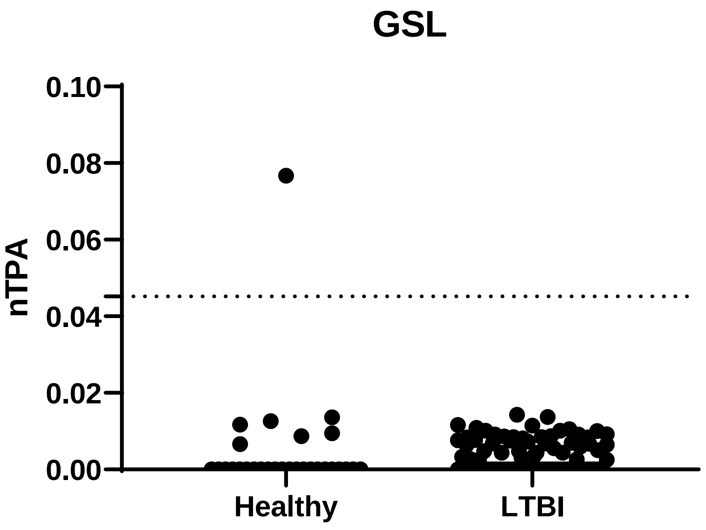
GSL
nTPA
0.10
0.08
0.06
0.04
0.02
0.00
Healthy
LTBI

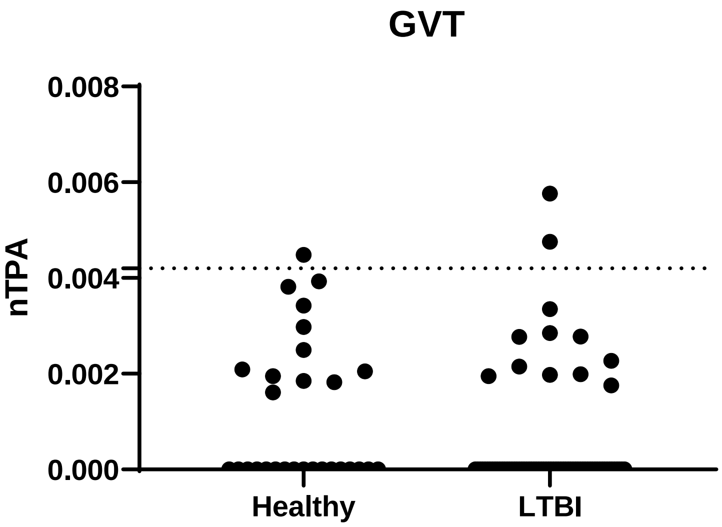
GVT
nTPA
0.008
0.006
0.004
0.002
0.000
Healthy
LTBI

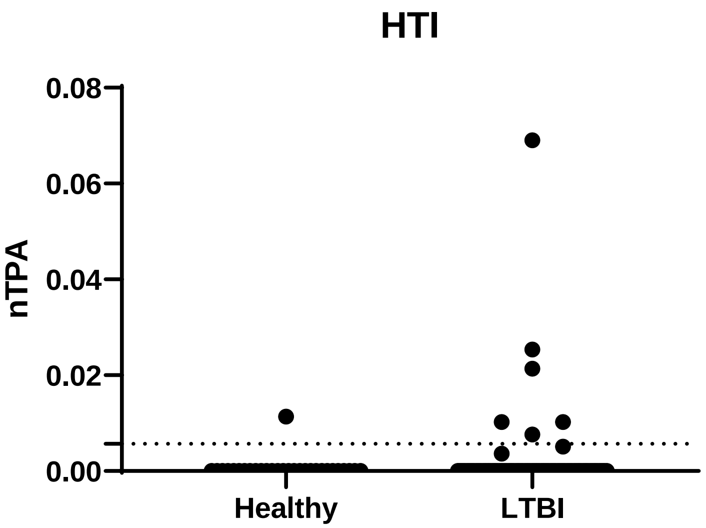
HTI
nTPA
0.08
0.06
0.04
0.02
0.00
Healthy
LTBI

h

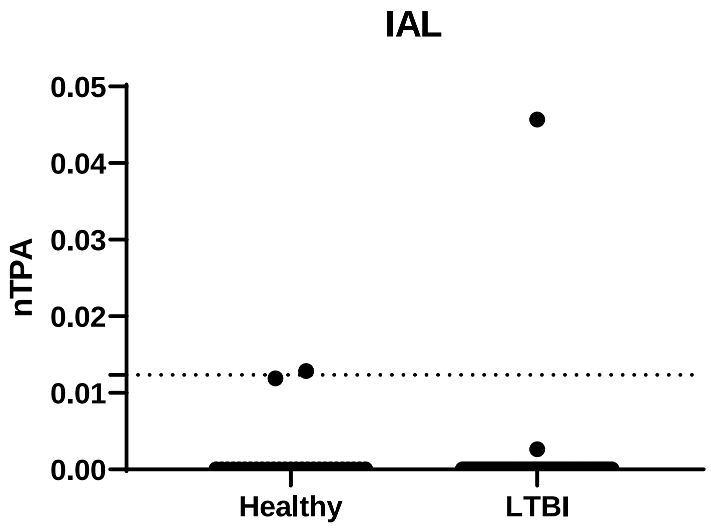
IAL
nTPA
0.05
0.04
0.03
0.02
0.01
0.00
Healthy
LTBI

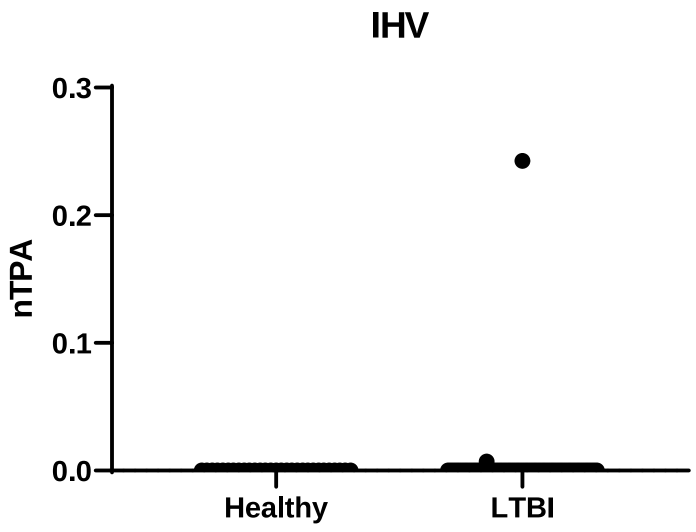
IHV
nTPA
0.3
0.2
0.1
0.0
Healthy
LTBI

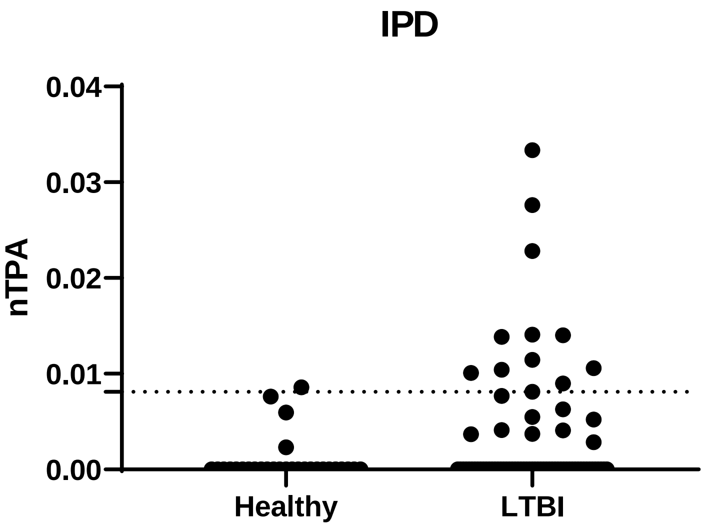
IPD
nTPA
0.04
0.03
0.02
0.01
0.00
Healthy
LTBI

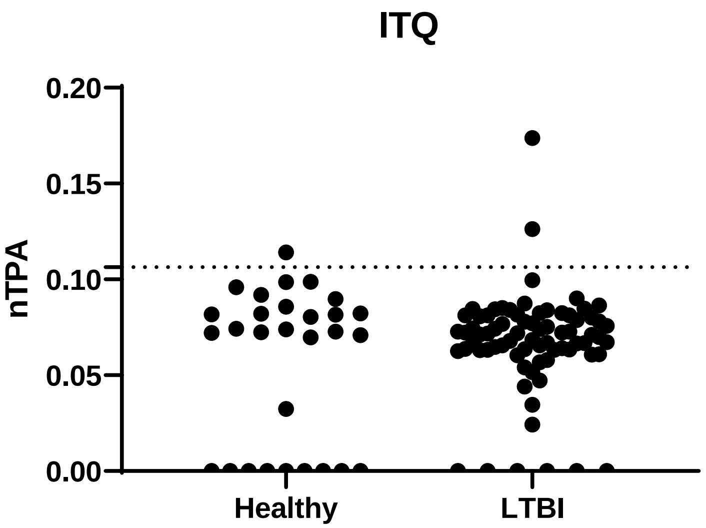
ITQ
nTPA
0.20
0.15
0.10
0.05
0.00
Healthy
LTBI

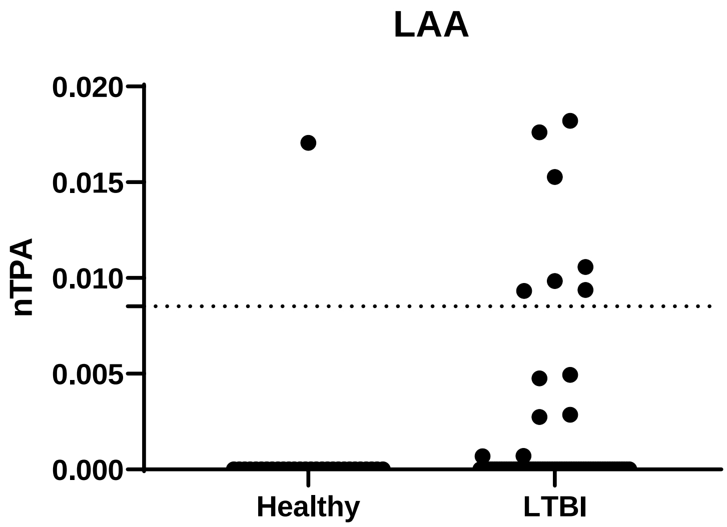

LAA
nTPA
0.020
0.015
0.010
0.005
0.000
Healthy
LTBI

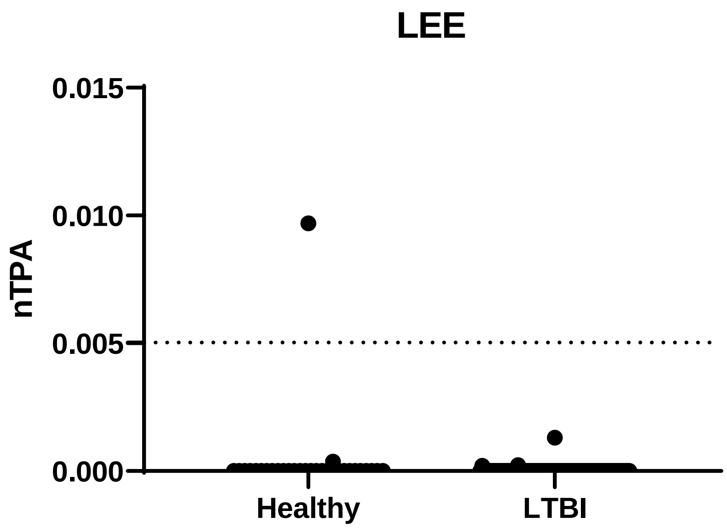

LEE
nTPA
0.015
0.010
0.005
0.000
Healthy
LTBI

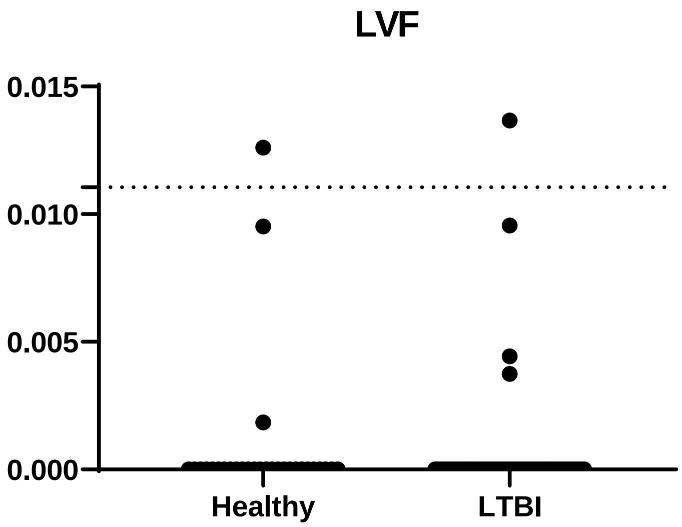
LVF
0.015
0.010
0.005
0.000
Healthy
LTBI

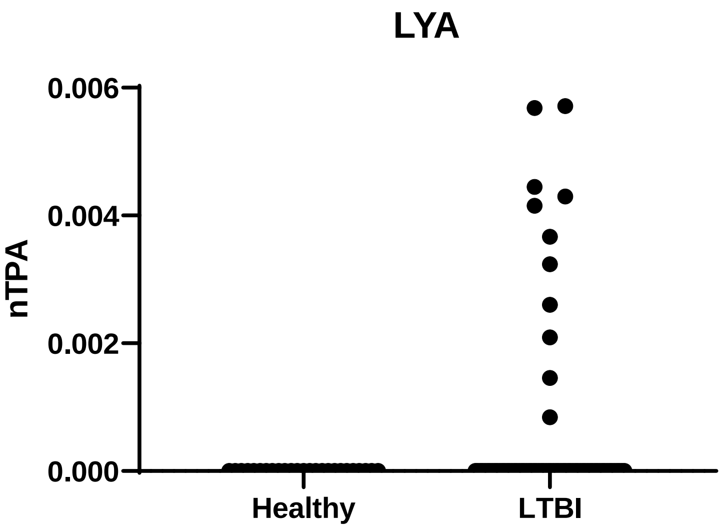
LYA
nTPA
0.006
0.004
0.002
0.000
Healthy
LTBI

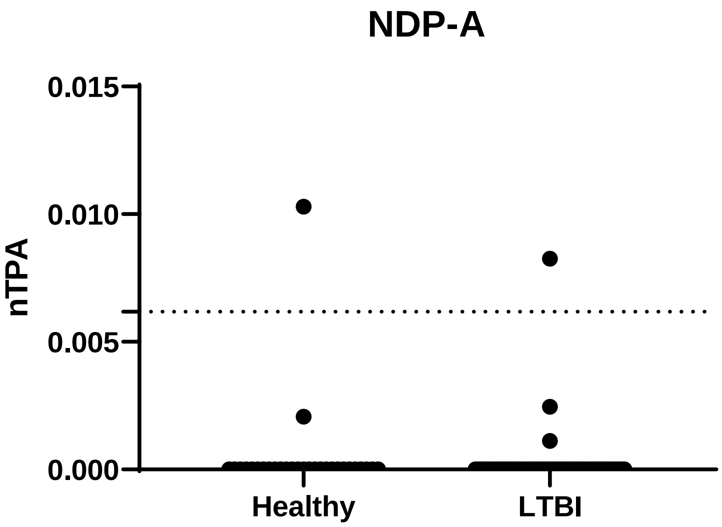
NDP-A
nTPA
0.015
0.010
0.005
0.000
Healthy
LTBI

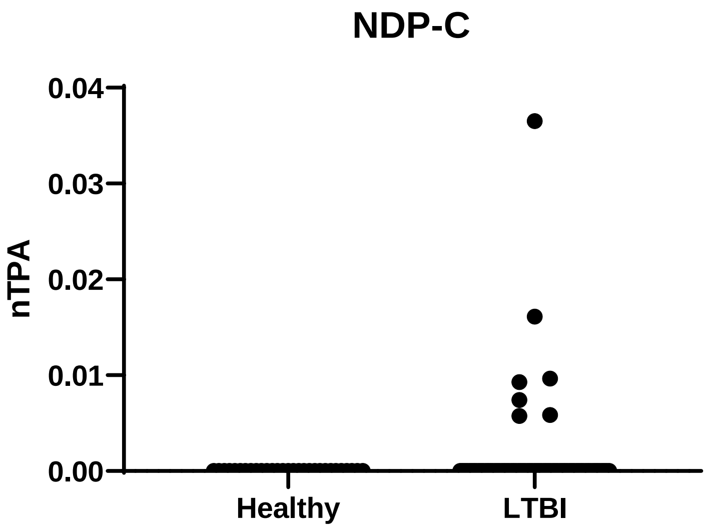
NDP-C
nTPA
0.04
0.03
0.02
0.01
0.00
Healthy
LTBI

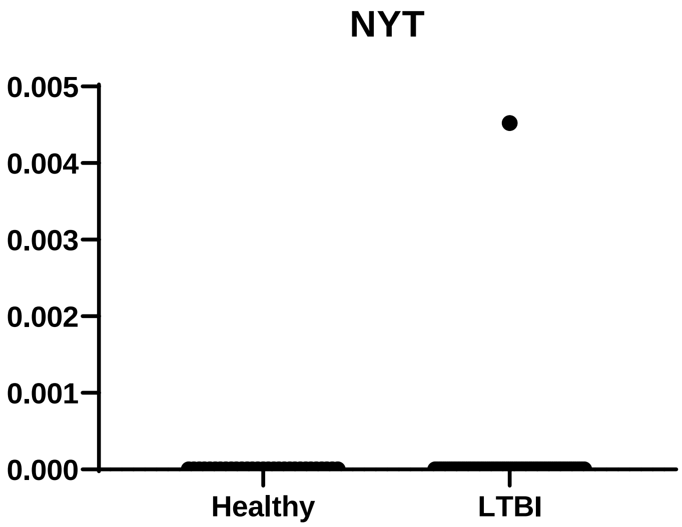
NYT
0.005
0.004
0.003
0.002
0.001
0.000
Healthy
LTBI

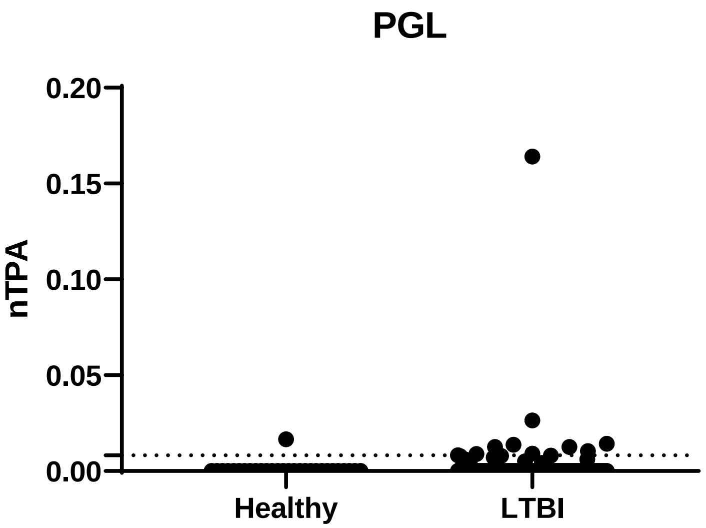
PGL
nTPA
0.20
0.15
0.10
0.05
0.00
Healthy
LTBI

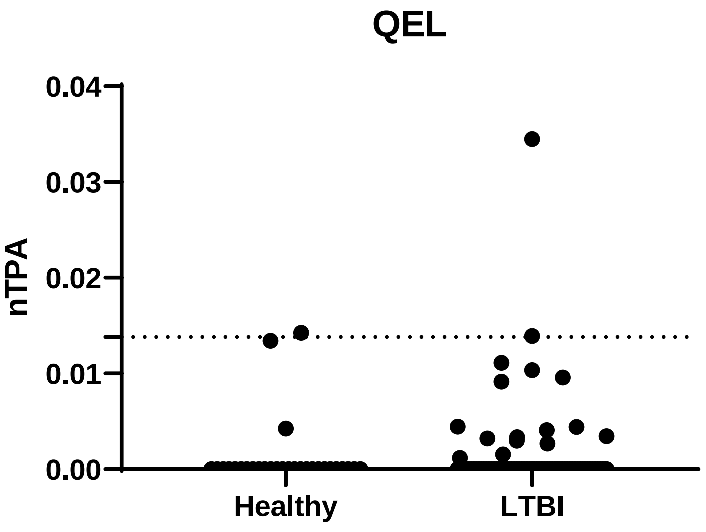
QEL
0.04
0.03
0.02
0.01
0.00
nTPA
Healthy
LTBI

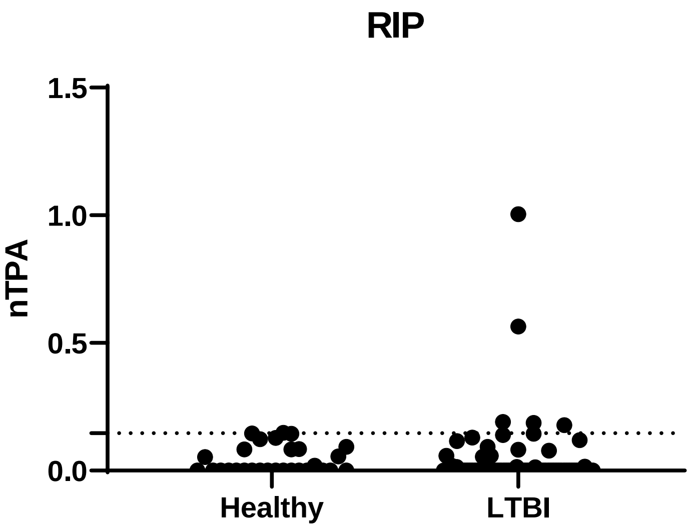
RIP
1.5
1.0
0.5
0.0
nTPA
Healthy
LTBI

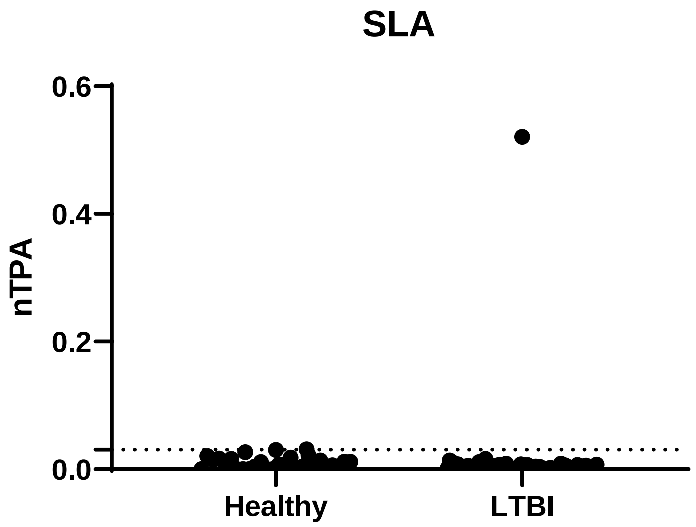
SLA
nTPA
0.6
0.4
0.2
0.0
Healthy
LTBI

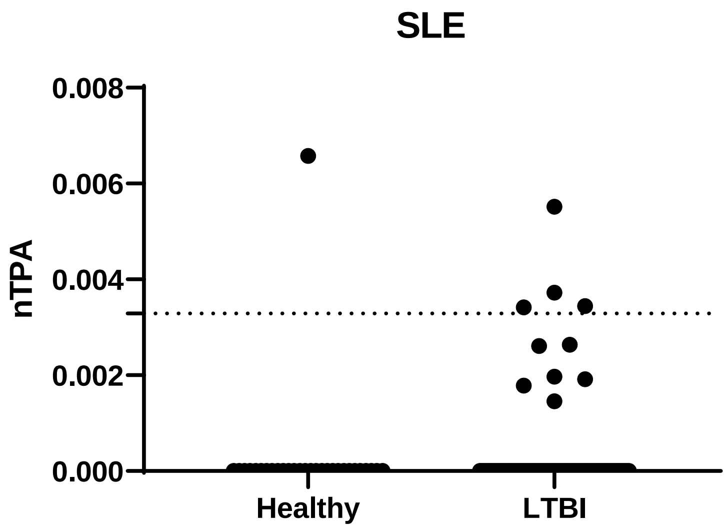
SLE
nTPA
0.008
0.006
0.004
0.002
0.000
Healthy
LTBI

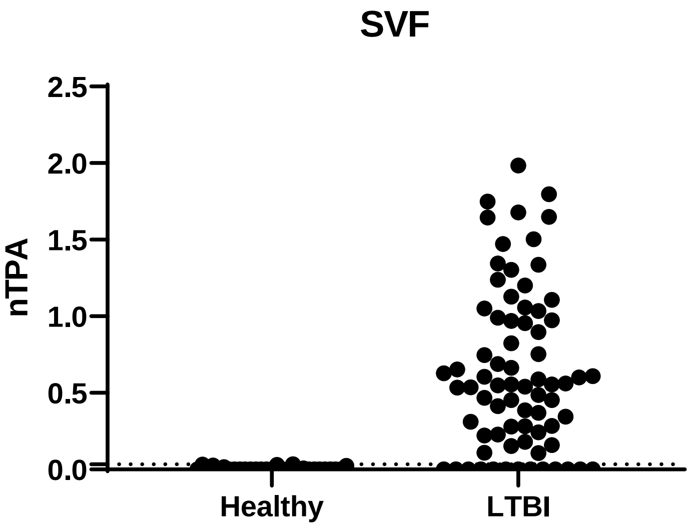
SVF
nTPA
2.5
2.0
1.5
1.0
0.5
0.0
Healthy
LTBI

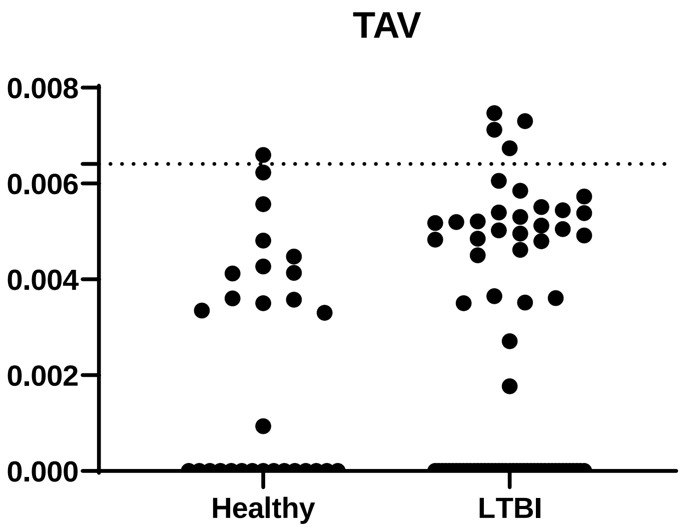
TAV
0.008
0.006
0.004
0.002
0.000
Healthy
LTBI

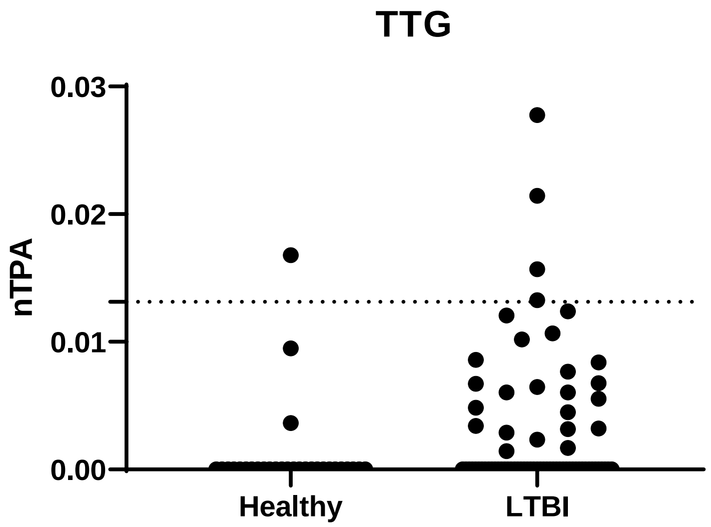
TTG
0.03
0.02
0.01
0.00
nTPA
Healthy
LTBI

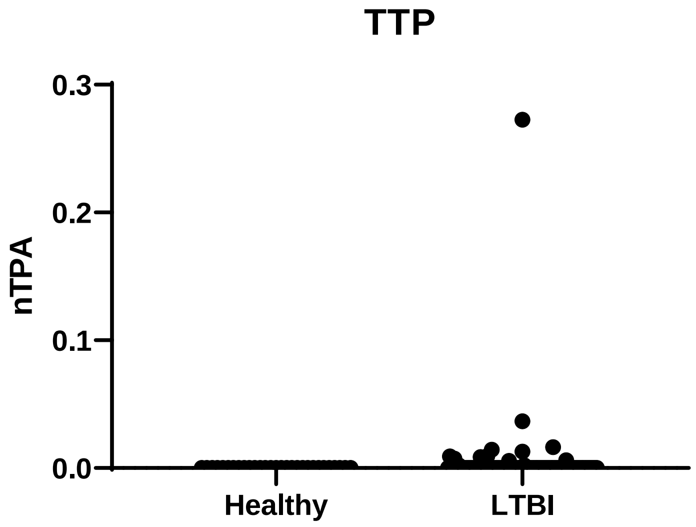
TTP
0.3
0.2
0.1
0.0
nTPA
Healthy
LTBI

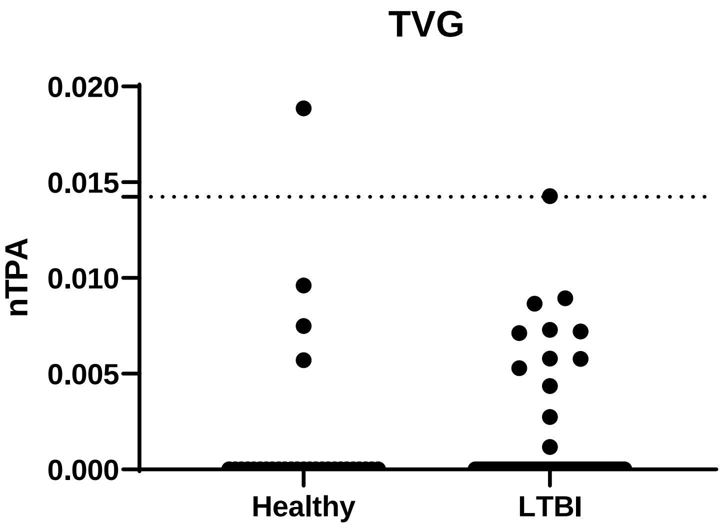
TVG
nTPA
0.020
0.015
0.010
0.005
0.000
Healthy
LTBI

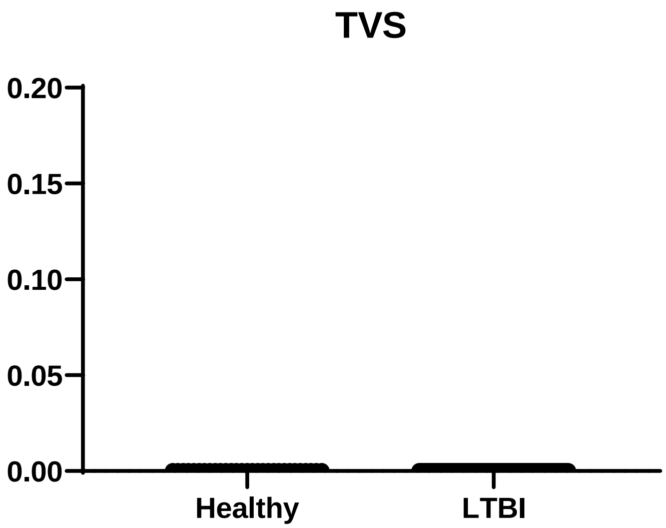
TVS
0.20
0.15
0.10
0.05
0.00
Healthy
LTBI

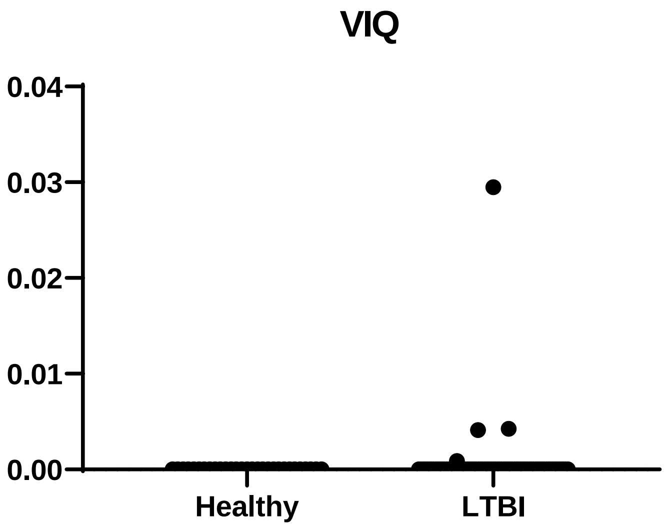
VIQ
0.04
0.03
0.02
0.01
0.00
Healthy
LTBI

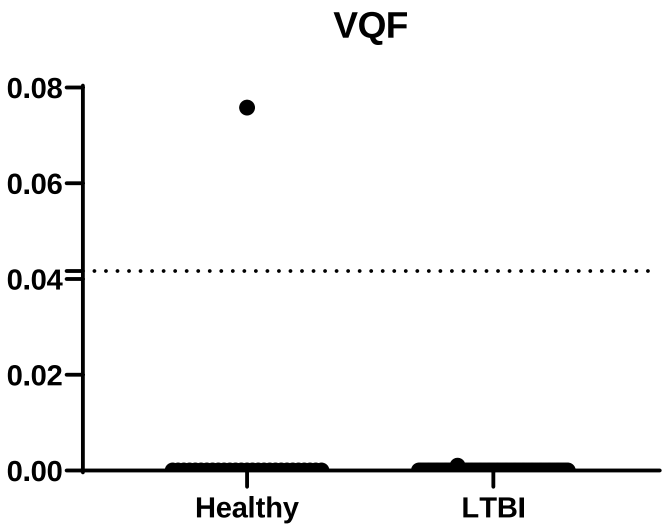
VQF
0.08
0.06
0.04
0.02
0.00
Healthy
LTBI

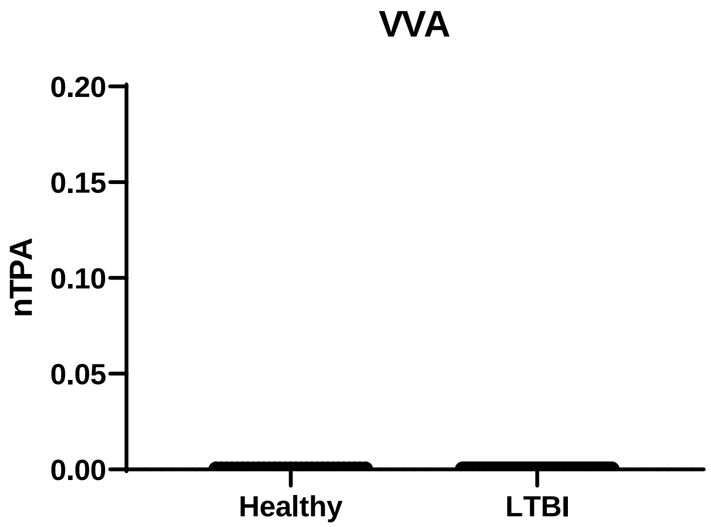
VVA
0.20
0.15
0.10
0.05
0.00
nTPA
Healthy
LTBI

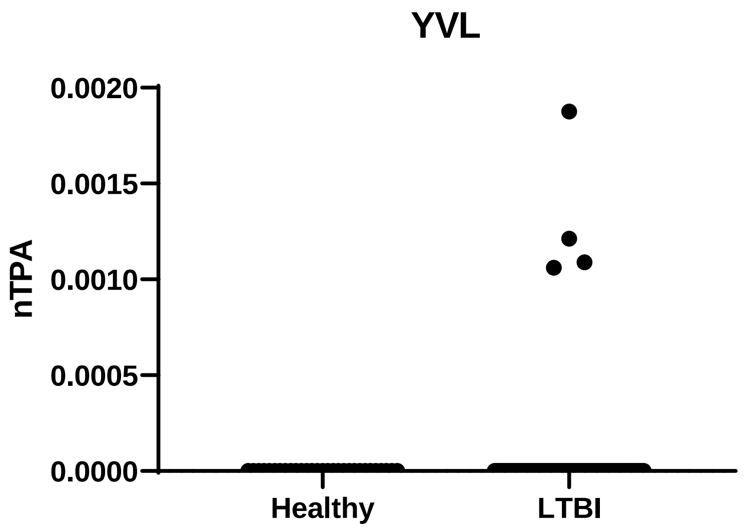
YVL
0.0020
0.0015
0.0010
0.0005
0.0000
nTPA
Healthy
LTBI
