# Supplementary material for: Identification of Mycobacterium tuberculosis Peptides in Serum Extracellular Vesicles from Persons with Latent Tuberculosis Infection
Source: J Clin Microbiol. 2020 May 26;58(6):e00393-20. doi: 10.1128/JCM.00393-20 (PMC7269374; doi:10.1128/JCM.00393-20)
Supplement: Supplemental file 3 [file JCM.00393-20-s0003.pdf]

Supplementary Material 3

Normalized Total Peak Area (nTPA) of healthy controls, individuals with latent tuberculosis infection (LTBI) and active tuberculosis (TB) for peptide SVF from GlnA1, DVL from GroES, FLL from GarA, and TTP and IPD from DnaK. TB patient samples correspond to those analyzed in reference 25. The dotted line corresponds to the calculated threshold of positivity (95th percentile of samples from health controls).

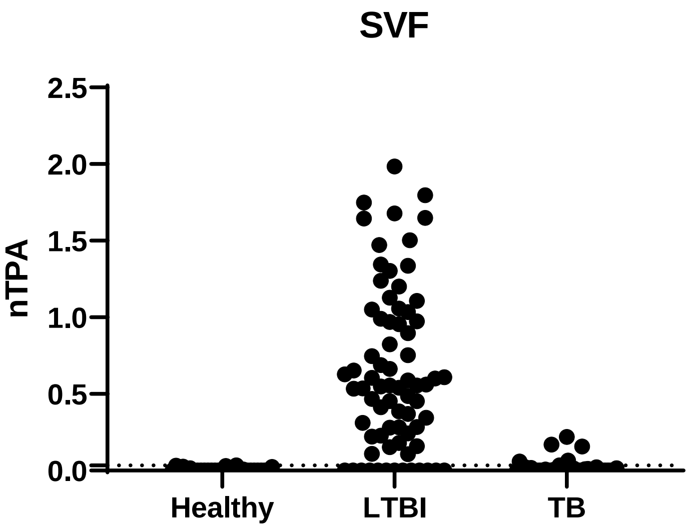

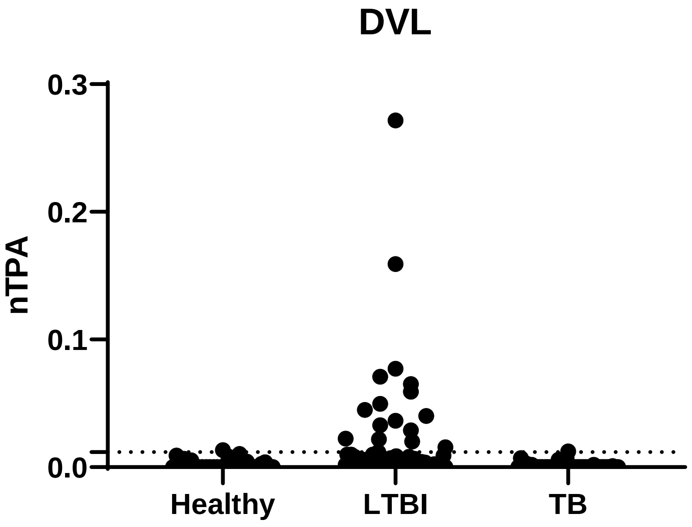

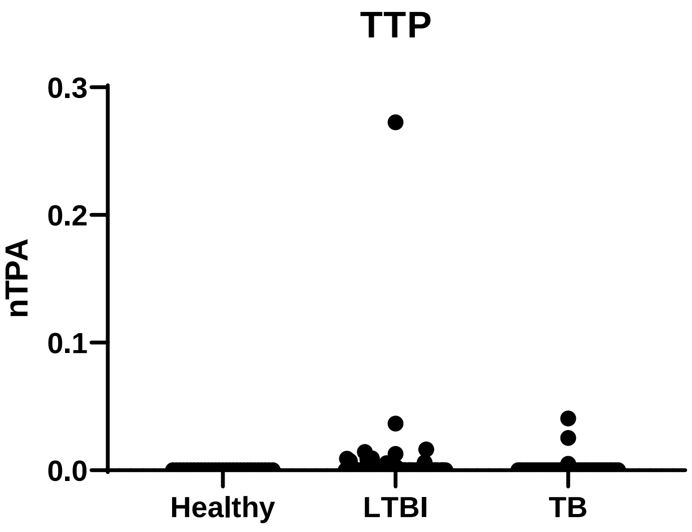
TTP
0.3
0.2
0.1
0.0
nTPA
Healthy
LTBI
TB

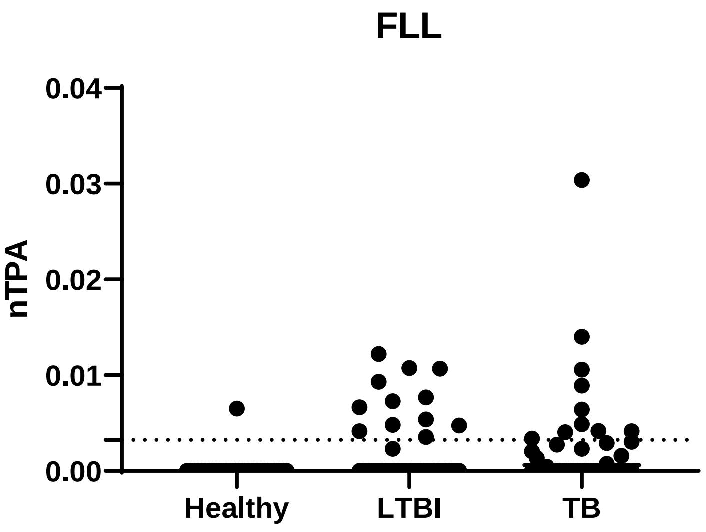
FLL
0.04
0.03
0.02
0.01
0.00
nTPA
Healthy
LTBI
TB

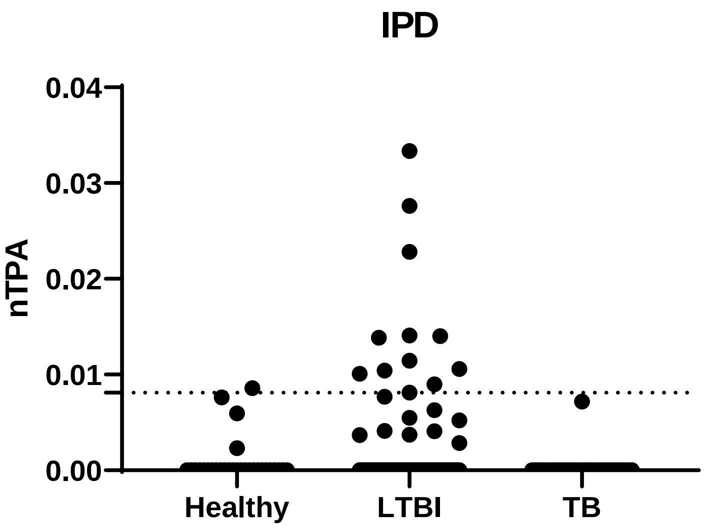
IPD
0.04
0.03
0.02
0.01
0.00
nTPA
Healthy
LTBI
TB

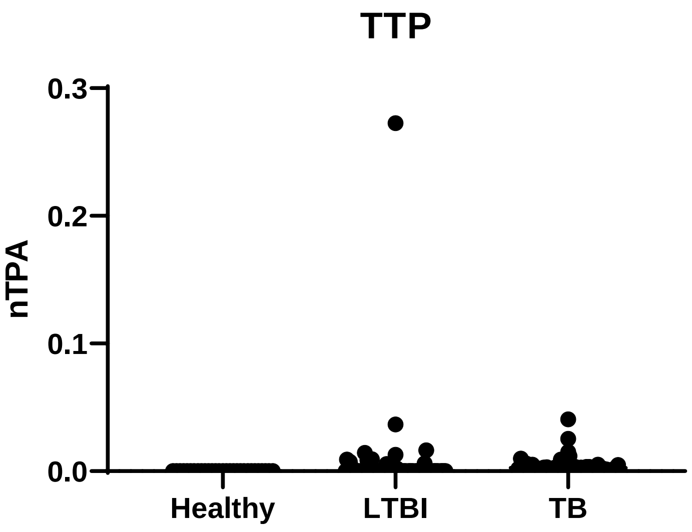
TTP
0.3
0.2
0.1
0.0
nTPA
Healthy
LTBI
TB
